# Novel HSA-PMEMA Nanomicelles Prepared via Site-Specific In Situ Polymerization-Induced Self-Assembly for Improved Intracellular Delivery of Paclitaxel

**DOI:** 10.3390/pharmaceutics17030316

**Published:** 2025-03-01

**Authors:** Yang Chen, Shuang Liang, Binglin Chen, Fei Jiao, Xuliang Deng, Xinyu Liu

**Affiliations:** 1 Institute of Medical Technology, Peking University Health Science Center, Beijing 100191, China; ychen@stu.pku.edu.cn; 2 Central Laboratory, NMPA Key Laboratory for Dental Materials, National Engineering Research Center of Oral Biomaterials and Digital Medical Devices, Peking University School and Hospital of Stomatology, Beijing 100081, China; 2416396054@bjmu.edu.cn (S.L.); 2311210516@stu.pku.edu.cn (B.C.); 2216387083@pku.edu.cn (F.J.)

**Keywords:** paclitaxel, nanoparticle, protein–polymer conjugate, self-assembly

## Abstract

**Background/Objectives**: Paclitaxel (PTX) is a potent anticancer drug that is poorly soluble in water. To enhance its delivery efficiency in aqueous environments, amphiphilic polymer micelles are often used as nanocarriers for PTX in clinical settings. However, the hydrophilic polymer segments on the surface of these micelles may possess potential immunogenicity, posing risks in clinical applications. To address this issue, nanomicelles based on human serum albumin (HSA)–hydrophobic polymer conjugates constructed via site-specific in situ polymerization-induced self-assembly (SI-PISA) are considered a promising alternative. The HSA shell not only ensures good biocompatibility but also enhances cellular uptake because of endogenous albumin trafficking pathways. Moreover, compared to traditional methods of creating protein–hydrophobic polymer conjugates, SI-PISA demonstrates higher reaction efficiency and better preservation of protein functionality. **Methods**: We synthesized HSA-PMEMA nanomicelles via SI-PISA using HSA and methoxyethyl methacrylate (MEMA)—a novel hydrophobic monomer with a well-defined and stable chemical structure. The protein activity and the PTX intracellular delivery efficiency of HSA-PMEMA nanomicelles were evaluated. **Results**: The CD spectra of HSA and HSA-PMEMA exhibited similar shapes, and the relative esterase-like activity of HSA-PMEMA was 94% that of unmodified HSA. Flow cytometry results showed that Cy7 fluorescence intensity in cells treated with HSA-PMEMA-Cy7 was approximately 1.35 times that in cells treated with HSA-Cy7; meanwhile, HPLC results indicated that, under the same conditions, the PTX loading per unit protein mass on HSA-PMEMA was approximately 1.43 times that of HSA. These collectively contributed to a 1.78-fold overall PTX intracellular delivery efficiency of HSA-PMEMA compared to that of HSA. **Conclusions**: In comparison with HSA, HSA-PMEMA nanomicelles exhibit improved cellular uptake and higher loading efficiency for PTX, effectively promoting the intracellular delivery of PTX. Tremendous potential lies in these micelles for developing safer and more efficient next-generation PTX formulations for tumor treatment.

## 1. Introduction

Cancer has increasingly become a critical issue for human health in modern society. Since 1937, it has been the second leading contributor to mortality in the U.S. [1]. Even during the COVID-19 pandemic in 2020–2021, the number of deaths caused by the virus was far lower than that of deaths due to cancer [2,3]. According to estimates, the U.S. is expected to experience 2,000,000 new cancer diagnoses in local people in 2024, with 600,000 expected cancer-related deaths [4].

In the list of FDA-approved anticancer drugs, paclitaxel (PTX)—a natural chemotherapy agent extracted from the bark of the Pacific yew tree—has long been the focus of research due to its excellent anticancer effects [5]. PTX is a microtubule-targeting agent that disrupts mitosis in cancer cells by promoting microtubule polymerization, leading to tumor cell apoptosis [6]. It is used to treat various cancers, including ovarian cancer [7], pancreatic cancer [8], non-small cell lung cancer [9], and breast cancer [10]. However, due to its low solubility in water, PTX often requires solubilizing delivery carriers for clinical applications. With the advancement of nanotechnology, delivering PTX via nanocarriers has emerged as a fascinating approach. Nanocarriers not only significantly increase PTX concentrations in aqueous environments but also enhance drug targeting to tumor tissues through the enhanced permeability and retention (EPR) effect [11]. Some researchers have developed various PTX nanocarriers based on inorganic materials, including gold nanoparticles [12], carbon nanotubes [13], and mesoporous silica nanoparticles [14]. While these nanocarriers demonstrate certain delivery capabilities for PTX, they exhibit notable drawbacks: carbon nanotubes and gold nanoparticles involve complex fabrication processes, and their costs are relatively high; porous silicon nanoparticles may experience premature drug leakage during delivery, along with potential hazards like promoting melanoma progression and inducing hemolysis [15].

In addition to inorganic materials, nanocarriers formed from organic amphiphilic molecules through self-assembly in aqueous solutions can also effectively deliver PTX [16,17,18]. Compared to inorganic carriers, these nanocarriers are more cost-effective [11] to produce and exhibit better biocompatibility [19,20], which has contributed to increasing interest from researchers in recent years, and several formulations based on amphiphilic molecules have already been approved for clinical use [11]. Specifically, these nanocarriers mainly include liposomes [21,22,23] and polymer micelles [24,25,26]. Liposomes, with a lipid bilayer structure similar to that of cell membranes, exhibit good biocompatibility and low immunogenicity in the human body. However, their stability is relatively poor, and their plasma half-life is rather short [27], which makes them subject to multiple limitations in clinical applications. In contrast, polymer micelles offer better stability, and their blood circulation time can be extended due to hydrophilic polymer shells (e.g., PEG); these hydrophilic polymer blocks can reduce nonspecific interactions between polymer micelles and blood components, as well as diminishing renal clearance by increasing the hydrodynamic volume of the micelles [28]. Nevertheless, these hydrophilic polymer shells may pose potential immunogenic risks [29,30,31,32], which raises safety concerns in the clinical applications of polymer micelles.

To build safer and more stable PTX nanocarriers, some researchers have proposed nanoparticle albumin-bound (nab^®^) technology and developed Abraxane^®^ (albumin-bound paclitaxel). This drug uses human serum albumin (HSA), a biocompatible protein that is widely present in blood, as a carrier for PTX. Through organic solvent emulsification and high-pressure homogenization, a cross-linked albumin shell is formed around the PTX core, creating nanoparticles with a hydrodynamic diameter of approximately 130 nm [33]. These nanocarriers not only exhibit good biocompatibility but also possess a certain capability of cell entry by means of the endogenous albumin transport pathway. However, the fabrication process involved in this method is relatively complex and costly. Moreover, it can lead to the denaturation of HSA, reducing the albumin shell’s ability to bind to cell surface receptors, which in turn limits the nanocarrier’s cellular uptake [34].

By combining the advantages of nab^®^ technology—such as improved biocompatibility and cellular uptake—with the inherent benefits of polymer micelles, we propose a superior strategy: constructing nanomicelles based on HSA–hydrophobic polymer conjugates for PTX delivery. To synthesize such nanomicelles, recent studies have introduced a powerful platform—site-specific in situ polymerization-induced self-assembly (SI-PISA) [35,36,37,38,39]. This method mainly consists of two steps: first, an atom transfer radical polymerization (ATRP) initiator is conjugated site-specifically to the hydrophilic protein to obtain the macroinitiator; second, via in situ ATRP, water-soluble monomers are polymerized onto the macroinitiator and transformed into a hydrophobic polymer, forming an amphiphilic protein–polymer conjugate that is able to self-assemble into various nanostructures in the aqueous solution. Notably, different from the traditional method used to construct protein–hydrophobic polymer conjugates [40,41,42], SI-PISA effectively enhances the reaction efficiency by avoiding steric hindrance between two large molecules [43]. What is more important, SI-PISA enables better control over the protein modification sites and the polymer’s molecular weight, and the entire process is solvent- and emulsifier-free, which preserves the protein’s activity effectively [35], addressing the inherent issue of protein denaturation in nab^®^ technology.

**Figure 1:**
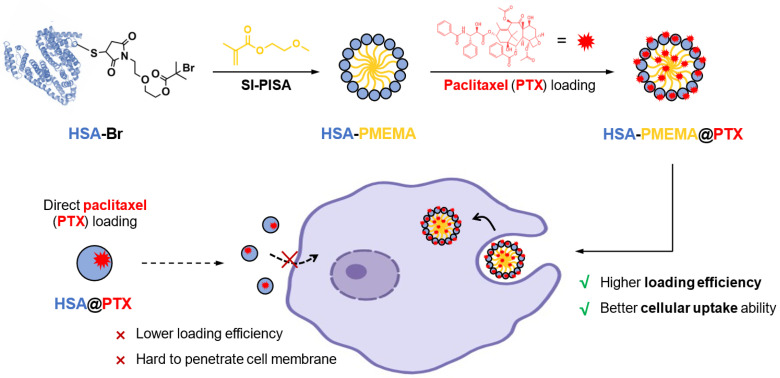
Scheme of novel HSA-PMEMA nanomicelles prepared via site-specific in situ polymerization-induced self-assembly (SI-PISA) for the improved intracellular delivery of paclitaxel. The blue spheres represent HSA, the yellow lines represent PMEMA, and the red explosion-shaped icons represent PTX. The purple cells are indicative of tumor cells. The solid lines illustrate the synthetic pathway of HSA-PMEMA@PTX and its interactions with the cells, while the dashed lines depict that of HSA@PTX.

In this study, we developed HSA-PMEMA nanomicelles via SI-PISA using HSA and methoxyethyl methacrylate (MEMA)—a novel PEG-like monomer. This monomer offers several unique advantages compared to other commonly used monomers in SI-PISA: unlike the most commonly used monomer, 2-hydroxypropyl methacrylate (HPMA) [35,38,39], MEMA has a well-defined chemical structure, which enhances batch-to-batch stability and is beneficial for quality control; in contrast to tertiary amine-based monomers [36,37], the hydrophilicity/hydrophobicity of PMEMA is less sensitive to pH fluctuations, ensuring better stability of the protein–polymer conjugate. The obtained HSA-PMEMA micelles exhibit a narrow size distribution and can largely maintain the activity of HSA. Notably, compared to HSA, the HSA-PMEMA micelles not only demonstrate improved cellular uptake by tumor cells but also can load PTX more efficiently, thereby promoting the overall effectiveness of PTX intracellular delivery to tumor cells (Figure 1).

## 2. Materials and Methods

### 2.1. Materials

Unless otherwise specified, all chemical reagents used in this study were purchased from Sigma Aldrich (St. Louis, MO, USA). Methoxyethyl methacrylate (MEMA) was purified by passing through an alkaline alumina column to remove the inhibitor before the reaction. Deuterated reagents and 4-nitrophenyl acetate were purchased from RHAWN (Shanghai, China). Recombinant human serum albumin (HSA) was obtained from Wuhan Healthgen Biotechnology Corp. (Wuhan, China, Catalog No. HYC002C01). A BCA assay kit was purchased from Thermo Scientific (Waltham, MA, USA, Catalog No. 23225). Sulfo-Cy7-NHS was obtained from DuoFluor (Shanghai, China, Catalog No. D10018). Defibrinated sheep blood was purchased from Solarbio (Beijing, China, Catalog No. TX0030), and erythrocyte lysis buffer was obtained from Beyotime (Shanghai, China, Catalog No. C3702). Nile red was purchased from Aladdin (Shanghai, China, Catalog No. N121291).

### 2.2. Synthesis

#### 2.2.1. Synthesis of DEEBMP

In an ice water bath, 200 mg of 1-(2-(2-hydroxyethoxy)ethyl)-1H-pyrrole-2,5-dione (HEPD) was dissolved in 10 mL of anhydrous dichloromethane, followed by the addition of 273 μL of triethylamine. Then, 497 μL of 2-bromo-2-methylpropionyl bromide (BMPB) was dissolved in 5 mL of anhydrous dichloromethane and slowly added to the reaction mixture over 15 min while maintaining the ice water bath. Once the addition of BMPB was finished, the ice water bath was taken away, allowing the reaction mixture to react overnight at room temperature. The resulting mixture was concentrated by rotary evaporation and subsequently purified by silica gel column chromatography (mobile phase: petroleum ether/ethyl acetate = 1/1, *v*/*v*) to obtain the product 2-(2-(2,5-dioxo-2,5-dihydro-1H-pyrrol-1-yl)ethoxy)ethyl 2-bromo-2-methylpropanoate (DEEBMP).

#### 2.2.2. Synthesis of HSA-Br

First, 66 mg of HSA powder was dissolved in 10 mL of Tris·HCl buffer solution (50 mM, containing 150 mM NaCl, pH = 7.4), followed by the addition of 1.7 mg of DEEBMP (previously dissolved in 200 μL of DMF). The reaction was conducted at room temperature for 2 h, followed by purification using a desalting column to obtain the product HSA-Br. The product was then concentrated by ultrafiltration and stored at −80 °C.

#### 2.2.3. Synthesis of HSA-PMEMA

A 1 mL PBS solution (with 10% glycerol) containing 12 mg of HSA-Br and 25 mg of MEMA was added to a Schlenk tube, and nitrogen was bubbled through to remove oxygen in the solution. Meanwhile, 4.7 mg of CuCl, 22.0 mg of CuCl_2_, and 37.9 mg of 1,1,4,7,10,10-hexamethyltriethylene tetramine (HMTETA) were added to 5 mL of Milli-Q (Merck, Darmstadt, Germany) water, which was then transferred to another container and bubbled with nitrogen to discharge oxygen. After bubbling, 400 μL of the catalyst solution was transferred to the Schlenk tube via a connecting tube. The Schlenk tube was then sealed, allowing the mixture to react in an atmosphere of nitrogen at 4 °C for 2 h. The reaction was subsequently quenched by exposure to air. Afterward, the product, HSA-PMEMA, was purified with size exclusion chromatography.

#### 2.2.4. Synthesis of HSA-PMEMA-Cy7 and HSA-Cy7

To 1 mL solutions containing 2.48 mg of HSA-PMEMA or HSA (measured by BCA assay) in 10 mM PBS buffer (pH = 7.4), 0.2569 mg of sulfo-Cy7-NHS (pre-dissolved in 5.14 μL DMSO) was added. The mixture was allowed to react at room temperature for 3 h. The reaction solution was then thoroughly dialyzed in 10 mM PBS buffer (MWCO 10 kDa) at 4 °C to obtain PBS solutions of HSA-PMEMA-Cy7 and HSA-Cy7.

#### 2.2.5. Synthesis of HSA-PMEMA@PTX and HSA@PTX

First, 1.29 mg of paclitaxel (PTX, previously dissolved in 12.9 μL of DMSO) was added separately to 1 mL of HSA-PMEMA solution and HSA solution (10 mM PBS buffer, pH = 7.4), both containing 10 mg of HSA (measured by BCA assay). The mixtures were stirred at room temperature for 12 h. Subsequently, the reaction mixture was dialyzed in 10 mM PBS buffer (MWCO 10 kDa) at 4 °C to remove DMSO. After dialysis, the resulting product was centrifuged at 10,000 rpm for 10 min to eliminate unloaded PTX precipitate. The supernatant obtained after centrifugation was concentrated by ultrafiltration to obtain PBS solutions of HSA-PMEMA@PTX and HSA@PTX.

### 2.3. Characterization

#### 2.3.1. Characterization of DEEBMP

Proton Nuclear Magnetic Resonance (^1^H NMR): 5 mg of synthesized DEEBMP was dissolved in 600 μL of deuterated chloroform, and its ^1^H NMR spectrum was recorded using a Bruker (Billerica, MA, USA) Ultrashield 600 MHz NMR spectrometer (software: TopSpin 4.4.1).

Electrospray Ionization Mass Spectrometry (ESI-MS): Synthesized DEEBMP was dissolved in methanol and characterized using an AB Sciex (Framingham, MA, USA) TripleTOF 4600 mass spectrometer with a detection range of 100–500 *m*/*z*. The data were analyzed using Analyst v1.5.

#### 2.3.2. Characterization of HSA-Br

The HSA and HSA-Br solutions were fully dialyzed in MilliQ (Merck, Darmstadt, Germany) water and then lyophilized. The characterization of HSA and HSA-Br was performed using matrix-assisted laser desorption ionization time-of-flight mass spectrometry (MALDI-TOF MS, AB Sciex 5800, USA, using sinapic acid as the matrix), aiming to verify the conjugation of the initiator motif by detecting the change in the molecular weight of HSA-Br compared to that of HSA.

#### 2.3.3. Physicochemical Characterization of HSA-PMEMA

Proton Nuclear Magnetic Resonance (^1^H NMR): The synthesized HSA-PMEMA sample was thoroughly dialyzed in MilliQ water, freeze dried, and then dissolved in deuterated dimethyl sulfoxide. Its ^1^H NMR spectrum was recorded using a Bruker Ultrashield 600 MHz NMR spectrometer.

Size Exclusion Chromatography (SEC): The HSA and HSA-PMEMA samples were dialyzed in MilliQ water, lyophilized, and then dissolved in hexafluoroisopropanol (HFIP). The molecular weight (MW) and dispersity (*Đ*) of the samples were analyzed using size exclusion chromatography (SEC) equipment consisting of a Waters (Milford, MA, USA) 1515 isocratic HPLC pump, Waters 2414 refractive index detector, Waters 2707 autosampler, and PL HFIPgel column (MW between 200 and 2,000,000). Hexafluoroisopropanol containing 3 mg/mL CF_3_COOK was employed as an eluent with a flow rate of 0.5 mL/min 40 °C. Commercial poly(methylmethacrylate)s (PMMAs) were used as the calibration standards.

Sodium Dodecyl Sulfate–Polyacrylamide Gel Electrophoresis (SDS-PAGE): Samples were dissolved in Beyotime SDS-PAGE protein loading buffer (Catalog No.: P0298) and heated at 95 °C for 5 min before being loaded onto a precast 8% polyacrylamide gel (LabLead, Nanjing, China, LabPAGE 8%, 12 wells, Catalog No.: P00812) for electrophoresis. Electrophoresis was performed at 160 V in Tris-MOPS-SDS running buffer (LabLead, Catalog No.: T7205M). After electrophoresis, the polyacrylamide gel was stained with Coomassie Brilliant Blue Ultra-Fast Staining Solution (Beyotime, China, Catalog No.: P0017F).

Dynamic Light Scattering (DLS): The particle size distribution of HSA-PMEMA samples under different solution conditions was measured using a Malvern (Malvern, UK) ZETASIZER NANO ZSP instrument. The HSA concentration in the samples ranged from 0.125 to 1.5 mg/mL (quantified by the BCA method), and the solution pH range was 5–9. The measurements were conducted at a temperature of 25 °C. The obtained data were analyzed using Zetasizer v7.02 software.

Transmission Electron Microscopy (TEM): The nanostructure of HSA-PMEMA was observed using a JEOL (Tokyo, Japan) JEM-1400 plus transmission electron microscope. The sample was loaded onto carbon film-covered copper grids and negatively stained with a 2% (*w*/*v*) phosphotungstic acid solution. After drying at room temperature, the sample was observed on the microscope.

Characterization of Critical Micelle Concentration (CMC): 15 μL of Nile red stock solution (0.88 mg/mL in DMSO) was added to 33 mL of PBS solution (pH = 7.4) to obtain a final concentration of 1.25 μM Nile red solution. The solution was used to repeatedly dilute the sample to be tested, halving the concentration with each dilution. After incubating the diluted samples at 37 °C for 1 h, 100 μL of each sample was added to a 96-well plate. The fluorescence intensity of the samples was measured using a microplate reader. A plot of fluorescence intensity versus concentration was generated, and the concentration corresponding to the inflection point of the curve represented the CMC.

#### 2.3.4. Evaluation of Protein Activity of HSA-PMEMA

Circular Dichroism (CD): After thoroughly dialyzing the samples in MilliQ water, their concentration was adjusted to 0.15 mg/mL (HSA, determined by the BCA assay). The CD spectrum was measured on a circular dichroism spectrometer (Applied Photophysics, Leatherhead, UK, Chirascan plus), and the data were analyzed using CDNN software 2.1.

Evaluation of Esterase-Like Activity: HSA, HSA-Br, and HSA-PMEMA dissolved in 10 mM PBS were concentrated to 0.3 mg/mL (HSA, determined by the BCA assay) using ultrafiltration. Then, 195 μL of each protein solution was added to the wells of a 96-well plate, followed by the addition of 5 μL of 4-nitrophenyl acetate (PNPA, 8 mM, dissolved in PBS containing 20% *v*/*v* acetonitrile). The reaction was monitored at 400 nm using a microplate reader (Molecular Devices, San Jose, CA, USA, SpectraMax iD5) to determine the amount of p-nitrophenol produced after 3 h. The assay was performed in triplicate for each sample.

#### 2.3.5. Stability Evaluation of HSA-PMEMA

To evaluate the stability of HSA-PMEMA in PBS, the sample was allowed to stand for 2 h to 6 days and characterized at different time points using DLS and CD. For the stability evaluation in serum, HSA-PMEMA was incubated in 37 °C FBS (HSA final concentration: 0.5 mg/mL) for 2 h to 6 days and characterized at different time points using DLS.

#### 2.3.6. Evaluation of PTX Intracellular Delivery Efficiency of HSA-PMEMA

Cellular Uptake of HSA-PMEMA: Cal27 cells were purchased from Wuhan Pricella Biotechnology Co., Ltd. (Wuhan, China) and were cultured in DMEM (high-glucose) containing 10% fetal bovine serum (FBS) and 1% penicillin/streptomycin (reagents were all purchased from Pricella Biotechnology) under the conditions of 5% CO_2_ at 37 °C. The cellular uptake capability of HSA and HSA-PMEMA was analyzed using confocal laser scanning microscopy (CLSM). Cal27 cells were seeded at a density of 2 × 10^5^ cells/dish in confocal dishes and incubated for 24 h. The culture medium was then removed, and 1 mL of medium containing 200 μg/mL HSA-Cy7 or HSA-PMEMA-Cy7 (HSA, determined by the BCA assay) was added to the culture dish. After 2 h of incubation, the medium was aspirated. The remaining cells were washed with PBS 3 times, stained with FITC-phalloidin (Solarbio, China, Catalog No: CA1620) and DAPI (Solarbio, China, Catalog No: C0060), and then imaged by CLSM. To further quantify the cellular uptake capability of HSA and HSA-PMEMA, a flow cytometry analysis was performed. Cal27 cells were incubated with HSA-Cy7 or HSA-PMEMA-Cy7 in 12-well plates and stained under the conditions described above. Then the cells were trypsinized and resuspended in 200–300 μL PBS buffer. The fluorescence intensity of the cell suspension was measured using a flow cytometer (Beckman, Indianapolis, IN, USA, CytoFLEX) with an excitation wavelength of 647 nm and an emission wavelength of 670 nm.

PTX Loading Efficiency of HSA-PMEMA: First, 1 mL aliquots of the PBS solutions of the HSA@PTX or HSA-PMEMA@PTX (containing 10 mg of HSA, measured by the BCA assay) obtained in Section 2.2.5 were freeze dried for 48 h. Then, 1 mL of methanol was added to extract PTX. After high-speed centrifugation, the supernatant was transferred to an appropriate container and concentrated to 100 μL. After another high-speed centrifugation to remove insoluble substances, the concentrated extract solution was analyzed using HPLC (AB Sciex, USA, QTRAP 5500) based on a standard curve. The PTX loading efficiency of HSA and HSA-PMEMA was then calculated. HPLC analysis was performed using a Shimadzu (Kyoto, Japan) LC-20A high-performance liquid chromatograph with a Waters ACQUITY UPLC BEH C18 column. Water was used as mobile phase A and acetonitrile as mobile phase B, with the concentration of B in the mobile phase ranging from 30% to 95%. The flow rate was 0.2 mL/min, and detection was performed at a UV absorption wavelength of 227 nm. The PTX loading content and efficiency were calculated as follows (unless otherwise specified, the carrier mass refers only to the protein mass and does not include the polymer mass). The normalized PTX loading content and efficiency were based on HSA@PTX at 100%.

(1) $$\mathrm{PTX}\;\mathrm{Loading}\;\mathrm{Content}\;(\%)=\frac{\mathrm{Mass}\;\mathrm{of}\;\mathrm{Loaded}\;\mathrm{PTX}}{\mathrm{Mass}\;\mathrm{of}\;\mathrm{Loaded}\;\mathrm{PTX}+\mathrm{Mass}\;\mathrm{of}\;\mathrm{Carrier}}\times 100$$

(2) $$\mathrm{PTX}\;\mathrm{Loading}\;\mathrm{Efficiency}\;(\%)=\frac{\mathrm{Mass}\;\mathrm{of}\;\mathrm{Loaded}\;\mathrm{PTX}}{\mathrm{Total}\;\mathrm{Mass}\;\mathrm{of}\;\mathrm{PTX}\;\mathrm{Used}\;\mathrm{in}\;\mathrm{Loading}\;\mathrm{Process}}\times 100$$

PTX Intracellular Delivery Efficiency of HSA-PMEMA@PTX: Cal27 cells were seeded into culture dishes at a density of 1.8 × 10^6^ cells/dish and incubated for 24 h. After removing the culture medium, 3 mL of culture medium containing HSA@PTX or HSA-PMEMA@PTX (10 mg HSA, measured by the BCA assay, refer to Section 2.2.5) was added, and the cells were further incubated for 2 h. The culture medium was then aspirated, and the remaining cells were washed three times with PBS. After the digestion of cells, the cell suspension was transferred to a centrifuge tube, and the supernatant was removed by centrifugation, followed by the addition of 50 μL methanol. The cells were thoroughly lysed using an ultrasonic cell disruptor (SCIENTZ, Ningbo, China, SCIENTZ-IID). After high-speed centrifugation, the supernatant was collected and analyzed for PTX content by HPLC. The conditions and methods of HPLC analysis were the same as in Section 2.3.6 The intracellular PTX delivery efficiency for both HSA@PTX and HSA-PMEMA@PTX was calculated as follows (unless specified, the carrier mass refers only to the protein mass and does not include the polymer mass). The normalized intracellular PTX delivery efficiency was based on HSA@PTX as 100%.

(3) $$\mathrm{Intracellular}\;\mathrm{PTX}\;\mathrm{Delivery}\;\mathrm{Efficiency}\;(\%)=\frac{\mathrm{Mass}\;\mathrm{of}\;\mathrm{PTX}\;\mathrm{Delivered}\;\mathrm{into}\;\mathrm{Cells}}{\mathrm{Total}\;\mathrm{Mass}\;\mathrm{of}\;\mathrm{PTX}\;\mathrm{and}\;\mathrm{Carrier}\;\mathrm{Used}\;\mathrm{in}\;\mathrm{Delivery}\;\mathrm{Process}}\times 100$$

#### 2.3.7. Evaluation of Pharmacodynamics of HSA-PMEMA@PTX

Culture medium solutions containing different concentration gradients of PTX, HSA@PTX, and HSA-PMEMA@PTX were prepared (refer to Section 2.2.5), with the PTX concentration ranging from 0.00096 to 1875 nM and the HSA concentration ranging from 0.000223 to 434.7826 μg/mL. The 4T1 cells (purchased from Wuhan Pricella Biotechnology Co. Ltd.) were cultured in DMEM high-glucose medium supplemented with 10% fetal bovine serum and 1% penicillin/streptomycin (all reagents purchased from Pricella Biotechnology). The cells were maintained under 5% CO_2_ at 37 °C. The 4T1 cells were seeded into 96-well plates at a density of 2500 cells per well. After 24 h, the medium was removed and replaced with 100 μL of the pre-prepared culture medium solutions containing different concentration gradients of PTX, HSA@PTX, or HSA-PMEMA@PTX. Additionally, wells containing only culture medium or culture medium with cells were included as blank or negative controls, respectively. The cells were incubated for another 24 h, and cell proliferation was assessed using a CCK-8 kit (purchased from Solarbio, Catalog No. CA1210). Cell viability in the blank group was set to 0%, and in the negative control group, cell viability was set to 100%. Inhibition curves and the half-maximal inhibitory concentration (IC_50_) values for each drug were calculated by fitting the data.

#### 2.3.8. In Vitro Release Study of HSA-PMEMA@PTX

A solution of HSA@PTX or HSA-PMEMA@PTX (refer to Section 2.2.5) containing 100 mg of HSA was placed in a dialysis bag (molecular weight cutoff: 3000 Da) and immersed in 100 mL of PBS at 37 °C for 2 to 48 h. At various time points, 5 mL of the PBS solution was withdrawn (after the volume decreased, it was replenished to 100 mL with PBS). The withdrawn solution was then lyophilized, and the remaining PTX was extracted with methanol. The PTX content was determined using UV-Vis spectroscopy, from which the amount of PTX released was calculated.

#### 2.3.9. Pharmacokinetic Study of HSA-PMEMA

HSA and HSA-PMEMA were labeled with Cy7. Specifically, 0.782 mg sulfo-Cy7-NHS (dissolved in 156 μL DMSO) was added to 10 mL of HSA-PMEMA solution (15 mg HSA, quantified by the BCA method) and HSA solution (10 mM PBS, pH 7.4), and the reaction was carried out at room temperature for 5 h. The reaction mixture was dialyzed at 4 °C against 10 mM PBS (MWCO 10 kDa), followed by concentration using ultrafiltration, yielding HSA-PMEMA-Cy7 and HSA-Cy7 PBS solutions. Balb/c female mice (8 weeks old) were intravenously injected with HSA-Cy7 or HSA-PMEMA-Cy7 at a dose of 50 mg HSA/kg. Blood samples were collected at different time points (1–48 h post-injection) via the tail vein and centrifuged at 4000× *g* for 15 min, and the serum was analyzed for Cy7 fluorescence intensity. The concentration of HSA-Cy7 or HSA-PMEMA-Cy7 in the serum was quantified using a fluorescence intensity–HSA concentration standard curve.

#### 2.3.10. In Vivo Biodistribution Study of HSA-PMEMA

The tumor-bearing mice required for biodistribution studies were established by subcutaneously implanting tumors. Specifically, SCC7 cells were injected subcutaneously into 6-week-old female C3H mice (5 × 10^6^ cells/mouse). After 2 weeks of further maintenance to allow the tumors to grow sufficiently, the mice were ready for the biodistribution studies. HSA and HSA-PMEMA were labeled with Cy7 (the method is the same as that mentioned in Section 2.3.9), and tumor-bearing C3H female mice were intravenously injected with HSA-Cy7 or HSA-PMEMA-Cy7 at a dose of 50 mg HSA/kg. At 12 and 24 h after injection, the fluorescence intensity of Cy7 at the tumor site was measured using a small-animal imaging system to estimate the amount of HSA-Cy7 or HSA-PMEMA-Cy7. At 72 h after injection, the mice were sacrificed and dissected. The fluorescence intensity of Cy7 in the major organs and tumor tissues was measured using the same imaging system to determine the distribution of HSA-Cy7 or HSA-PMEMA-Cy7.

#### 2.3.11. Hemolysis Assay of HSA-PMEMA@PTX

PBS solutions containing 2.62 μg PTX/mL and 5.24 μg PTX/mL of free PTX, HSA@PTX, and HSA-PMEMA@PTX were prepared (refer to Section 2.2.5), along with PBS and red blood cell lysis solution as negative and positive controls, respectively. Then, 200 μL of defibrinated sheep blood was added to 1 mL of each solution. The mixtures were incubated at 37 °C for 30 min and then centrifuged at 2500 rpm for 5 min. The supernatant from each group was collected, and the absorbance at 450 nm was measured using a microplate reader. The hemolysis rate for each solution was calculated using the following formula.

(4) $$\mathrm{Hemolysis}\;\mathrm{Ratio}\;(\%)=\frac{\mathrm{Absorbance}\;\mathrm{of}\;\mathrm{Supernatant}\;\mathrm{of}\;\mathrm{the}\;\mathrm{Measured}\;\mathrm{Solution}}{\mathrm{Absorbance}\;\mathrm{of}\;\mathrm{Supernatant}\;\mathrm{of}\;\mathrm{the}\;\mathrm{Positive}\;\mathrm{Control}}\times 100$$

### 2.4. Statistics

Data obtained in this study were analyzed using GraphPad Prism 5 software. Statistical significance was evaluated using Student’s *t*-test, and the results are presented as mean ± standard deviation. The significance levels are denoted as follows: * *p* < 0.05, ** *p* < 0.01, and *** *p* < 0.001.

## 3. Results and Discussion

### 3.1. Synthesis and Characterization of HSA-Br

**Figure 2:**
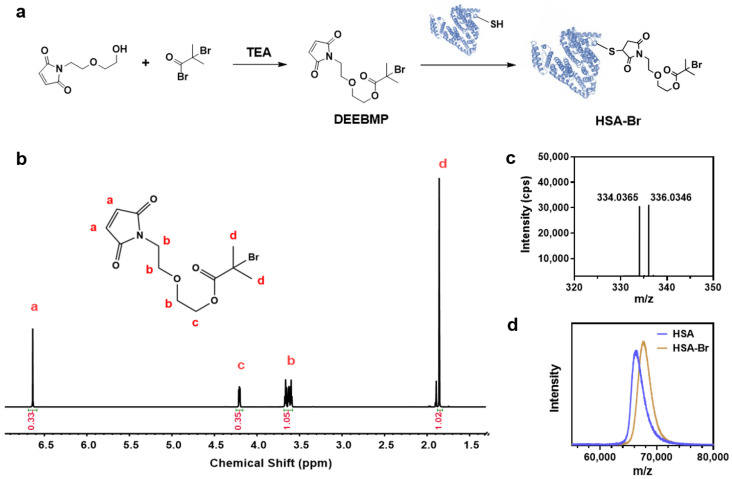
Synthesis and characterization of HSA-Br. (**a**) Scheme of the synthesis of HSA-Br. (**b**) ^1^H NMR spectrum of DEEBMP in CDCl_3_. Via the identification of peaks in the spectrum, we confirmed that the synthesized DEEBMP has a chemical structure consistent with our expectations. (**c**) ESI-MS spectrum of DEEBMP. The signals at *m*/*z* 334 and 336 correspond to the M + H^+^ signals of DEEBMP containing ^79^Br and ^81^Br, respectively, confirming the presence of DEEBMP (theoretical molecular weight: 334) in the synthesized product. (**d**) MALDI-TOF MS characterization of HSA and HSA-Br. Given that the theoretical molecular weight of HSA is approximately 66.5 kDa, the signal peaks in the figure correspond to z = 1. The *m*/*z* signal peak of HSA-Br is shifted to the right compared to that of HSA, indicating an increase in molecular weight, which confirms the successful conjugation of DEEBMP to HSA.

HSA contains 35 cysteine residues, 34 of which are stabilized by disulfide bonds, with only Cys 34 possessing a free thiol group [44]. This free thiol group theoretically allows for the site-specific modification of HSA. According to this, we synthesized the ATRP initiator DEEBMP, which contains a maleimide group at its terminus. Through the Michael addition reaction, DEEBMP was conjugated to the thiol group of Cys 34 in HSA site-specifically to obtain HSA-Br (Figure 2a). The synthesized DEEBMP was characterized by proton nuclear magnetic resonance (^1^H NMR) spectroscopy. In the ^1^H NMR spectrum (Figure 2b), all the signals corresponding to the hydrogen atoms in DEEBMP could be found, confirming that the chemical structure of DEEBMP matched the expected one. As a further verification, electrospray ionization mass spectrometry (ESI-MS) was employed for the characterization of DEEBMP. The result (Figure 2c) showed signal peaks at *m*/*z* 334 and 336, which correspond to the M + H^+^ signals of DEEBMP with ^79^Br and ^81^Br, respectively, confirming the presence of DEEBMP in the product (theoretical molecular weight of DEEBMP: 334 Da). Consequently, in order to determine whether DEEBMP was conjugated to HSA, we applied matrix-assisted laser desorption ionization time-of-flight mass spectrometry (MALDI-TOF MS) to analyze HSA and the synthesized HSA-Br. As the theoretical molecular weight of HSA is approximately 66.5 kDa, the *m*/*z* signal peak in the spectrum (Figure 2d) corresponds to z = 1. Obviously, the *m*/*z* signal for HSA-Br was shifted to the right compared to that for HSA, indicating a larger molecular weight of HSA-Br due to the successful conjugation of DEEBMP to HSA.

### 3.2. Synthesis and Physicochemical Characterization of HSA-PMEMA

**Figure 3:**
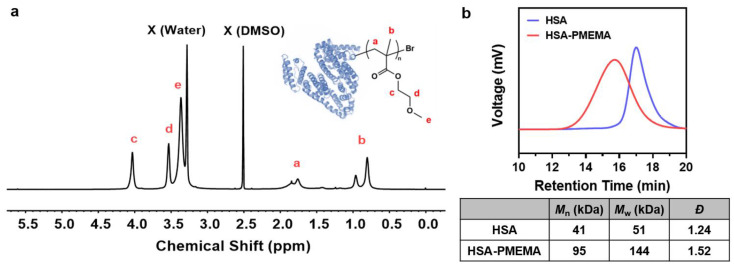
Physicochemical characterization of HSA-PMEMA. (**a**) ^1^H NMR spectrum of HSA-PMEMA in deuterated dimethyl sulfoxide. The figure shows the chemical structure of HSA-PMEMA. All proton signals corresponding to the PMEMA chain are observed in the spectrum, confirming the successful in situ growth of PMEMA on HSA. X (Water) represents the water peak, and X (DMSO) represents the solvent peak of DMSO. (**b**) GPC traces of HSA and HSA-PMEMA. The table shows the number-averaged molecular weight (*M*_n_), weight-averaged molecular weight (*M*_w_), and dispersity (*Đ*) of the samples.

**Figure 4:**
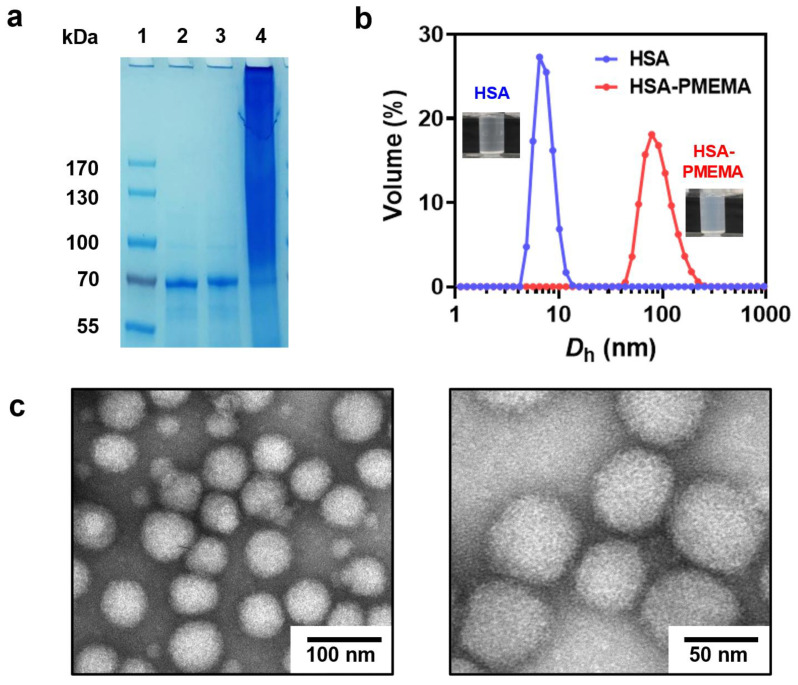
Physicochemical characterization of HSA-PMEMA. (**a**) SDS-PAGE results before and after the in situ growth of PMEMA on HSA. Lane 1: Protein marker; Lane 2: HSA; Lane 3: HSA-Br; Lane 4: HSA-PMEMA. (**b**) DLS results before and after the in situ growth of PMEMA on HSA. *D*_h_ represents the hydrodynamic diameter. The illustrations show the difference between the appearance of HSA and HSA-PMEMA in PBS buffer. (**c**) Representative TEM images of HSA-PMEMA micelles.

We employed site-specific in situ ATRP to grow PMEMA on HSA-Br, resulting in an amphiphilic HSA-PMEMA conjugate. The synthesized HSA-PMEMA was characterized by proton nuclear magnetic resonance (^1^H NMR) spectroscopy, and its spectrum (Figure 3a) showed peaks corresponding to all hydrogen atoms in the PMEMA segment; sodium dodecyl sulfate–polyacrylamide gel electrophoresis (SDS-PAGE) analysis of HSA, HSA-Br, and HSA-PMEMA was also performed (Figure 4a), and in the lane of HSA-PMEMA, a distinct new band with a higher molecular weight compared to that of HSA and HSA-Br was observed. These results all indicated the successful in situ growth of PMEMA on HSA-Br. To further characterize the molecular weight of the HSA-PMEMA conjugate, we performed size exclusion chromatography (SEC) in hexafluoroisopropanol using poly(methylmethacrylate)s (PMMAs) as the calibration standards (Figure 3b). The apparent weight-averaged molecular weight (*M*_w_) of HSA-PMEMA was 144 kDa, higher than that of HSA (51 kDa), further confirming the successful growth of PMEMA on HSA-Br. The dispersity (*Đ*) of HSA-PMEMA was 1.52, which indicates a relatively narrow molecular weight distribution, suggesting that the process of in situ polymerization was well controlled.

During the polymerization of HSA-PMEMA, we observed an increase in the opacity of the polymerization solution (Figure 4b), indicating the transition of water-soluble HSA to amphiphilic HSA-PMEMA, which formed nanostructures via in situ self-assembly [35]. To further confirm the occurrence of self-assembly, we characterized the resulting HSA-PMEMA emulsion using dynamic light scattering (DLS) and transmission electron microscopy (TEM). DLS analysis (Figure 4b) showed that the Z-average diameter of the HSA-PMEMA particles was 99.1 nm, significantly larger than that of the HSA particles, with a monomodal distribution and a polydispersity index (PdI) of 0.108. This suggests that HSA-PMEMA self-assembled into nanomicelles with a relatively narrow size distribution in aqueous solution. Additionally, TEM images revealed spherical nanoparticles approximately 50 nm in diameter with a relatively uniform size distribution, reinforcing the confirmation of the self-assembly of the HSA-PMEMA.

### 3.3. Evaluation of Protein Activity

**Figure 5:**
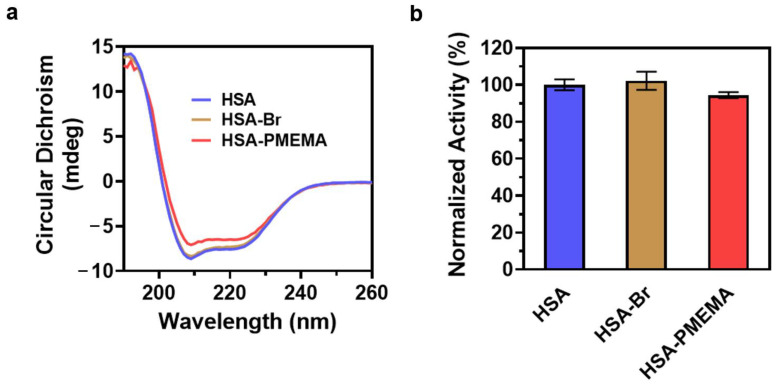
Evaluation of the protein activity of HSA-PMEMA. (**a**) CD spectra of HSA, HSA-Br, and HSA-PMEMA. The similar shapes of the CD spectra indicate that the in situ growth of PMEMA on HSA does not affect its secondary structure. (**b**) Normalized lipase-like activity of HSA, HSA-Br, and HSA-PMEMA.

Protein activity is closely related to secondary structure, and changes in the secondary structure can lead to a reduction in or loss of protein activity [45,46,47]. To examine whether the secondary structure of HSA was altered during the preparation of HSA-PMEMA, we performed circular dichroism (CD) spectroscopy on HSA, HSA-Br, and HSA-PMEMA. The CD spectra of all three samples (Figure 5a) exhibited similar shapes, with a characteristic doublet at ~209/222 nm, which indicates that the secondary structure of HSA remained largely unchanged during the process of SI-PISA, beneficial for the preservation of the activity of HSA.

Studies have shown that HSA exhibits esterase-like activity, capable of hydrolyzing various small-molecule esters, including p-nitrophenyl acetate (PNPA) [48]. To further quantify the esterase-like activity of HSA during the preparation of HSA-PMEMA, we used PNPA as a substrate and measured the amount of p-nitrophenol produced over 3 h by HSA, HSA-Br, and HSA-PMEMA through UV-Vis spectrophotometry. The result (Figure 5b) showed that the relative activity of HSA (consistent with the relative amount of released p-nitrophenol) in HSA-Br and HSA-PMEMA was 102% and 94%, respectively, that of unmodified HSA, with no obvious difference observed. This suggests that in the process of SI-PISA to construct HSA-PMEMA, the esterase-like activity of HSA was effectively preserved.

### 3.4. Evaluation of PTX Intracellular Delivery Efficiency and Pharmacodynamics

As we expected, HSA-PMEMA can exhibit enhanced efficiency in delivering PTX into tumor cells compared to HSA. This can be attributed to two main reasons. First, HSA-PMEMA forms nanomicelles with a hydrodynamic diameter (*D*_h_) of approximately 50 nm, which is more favorable for cellular uptake compared to HSA (*D*_h_ < 10 nm) [49]. Second, the hydrophobic polymer block in HSA-PMEMA allows for additional PTX loading, resulting in a higher PTX payload per unit mass of protein under the same conditions.

**Figure 6:**
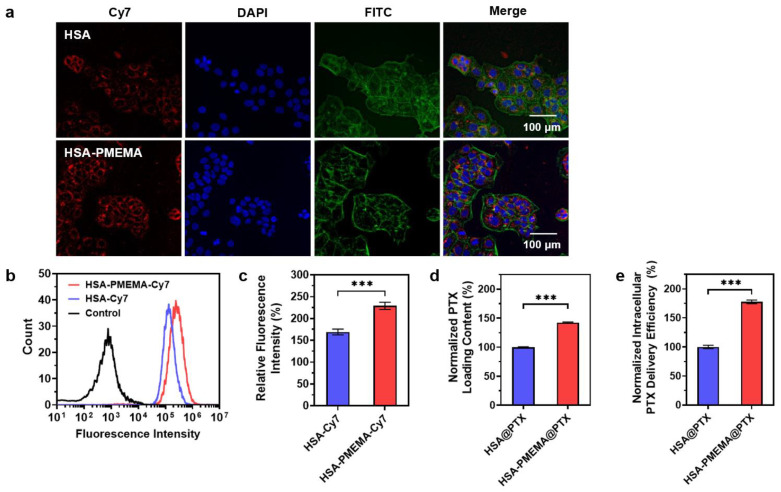
Evaluation of the PTX intracellular delivery efficiency of HSA-PMEMA. (**a**) CLSM images of Cal27 cells co-cultured with HSA-Cy7 or HSA-PMEMA-Cy7 for 2 h. In the CLSM images, green, blue, and red fluorescence correspond to FITC (cytoskeleton), DAPI (nucleus), and Cy7 (HSA or HSA-PMEMA), respectively. (**b**) Flow cytometry analysis of Cal27 cells co-cultured with HSA-Cy7 or HSA-PMEMA-Cy7. (**c**) Relative fluorescence intensity of Cal27 cells co-cultured with HSA-Cy7 or HSA-PMEMA-Cy7 compared to the control group (n = 3). The result was derived from flow cytometry analysis. (**d**) Normalized PTX loading content of HSA@PTX and HSA-PMEMA@PTX. (**e**) Normalized intracellular PTX delivery efficiency of HSA@PTX and HSA-PMEMA@PTX. *** represents *p* < 0.001.

To verify the cellular uptake efficiency of HSA and HSA-PMEMA, we labeled both samples with Cy7 and co-cultured them with model cells for 2 h under identical conditions. In this study, Cal27 cells, which are responsive to PTX and have substantial value in clinical research [50], were used as the model cells. The cellular uptake was then assessed via confocal laser scanning microscopy (CLSM) and flow cytometry. CLSM images (Figure 6a) showed stronger red fluorescence (Cy7) in cells treated with HSA-PMEMA-Cy7 than in those treated with HSA-Cy7. For further quantification, flow cytometry results (Figure 6b,c) also showed that the Cy7 fluorescence intensity in cells treated with HSA-PMEMA-Cy7 was approximately 1.35 times that in cells treated with HSA-Cy7. These results collectively indicate that HSA-PMEMA is more efficiently internalized by Cal27 cells compared to HSA, consistent with our previous expectations.

To evaluate the PTX loading capacity per unit mass of protein, we mixed PTX with HSA or HSA-PMEMA under identical conditions and then removed the unloaded PTX to obtain HSA@PTX and HSA-PMEMA@PTX. The PTX loaded in both samples was extracted using methanol and quantified by high-performance liquid chromatography (HPLC). The HPLC results (Figure 6d) showed that, under the same conditions, the PTX loading per unit protein mass on HSA-PMEMA was approximately 1.43 times that of HSA, which corresponds to our earlier expectations.

With the aim of directly evaluating the PTX intracellular delivery efficiency of HSA and HSA-PMEMA into tumor cells, we co-cultured equal protein amounts of HSA@PTX or HSA-PMEMA@PTX with Cal27 cells under the same conditions for 2 h. Afterward, the intracellular PTX was extracted using methanol and quantified by high-performance liquid chromatography (HPLC). The HPLC results (Figure 6e) showed that the amount of PTX delivered via HSA-PMEMA@PTX into Cal27 cells was approximately 1.78 times higher than that via HSA@PTX, indicating that HSA-PMEMA facilitated more efficient PTX delivery into cells compared to HSA. Moreover, it is noteworthy that the overall enhancement in intracellular PTX delivery by HSA-PMEMA was greater than the individual contributions of the two factors—cellular uptake ability and PTX loading capacity—individually mentioned earlier. This further suggests that the improved PTX delivery efficiency of HSA-PMEMA results from the combined effects of both aforementioned factors.

**Figure 7:**
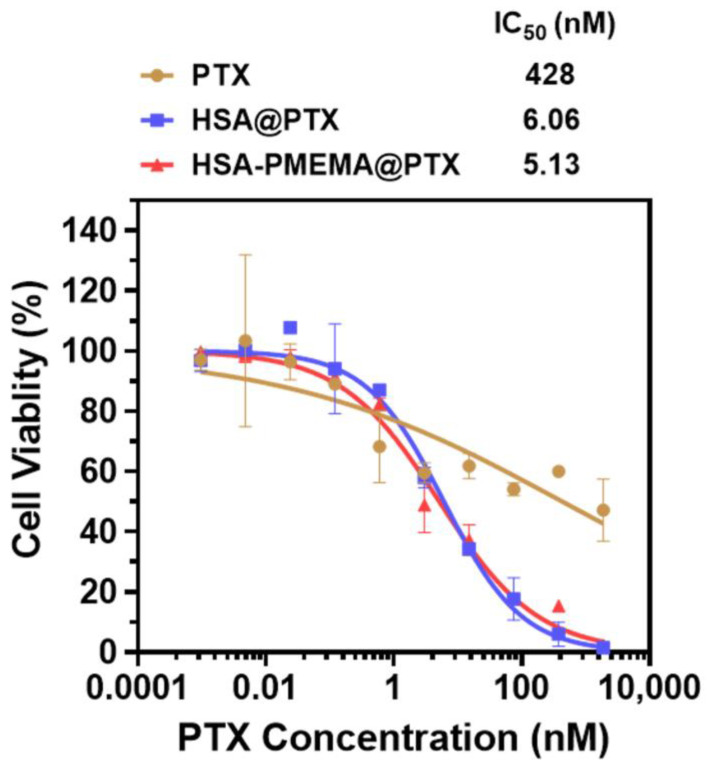
Anticancer activity of HSA@PTX and HSA-PMEMA@PTX against 4T1 cells (measurement was based on PTX concentration). The half-maximal inhibitory concentration (IC_50_) was calculated from the inhibition curves fitted.

To evaluate the antitumor efficacy of HSA-PMEMA@PTX benefitting from the improved intracellular delivery efficiency of PTX, we conducted pharmacological assessments of HSA@PTX and HSA-PMEMA@PTX. For this purpose, we selected 4T1 cells, a tumor cell line with significant clinical relevance, as the model to measure the therapeutic effect. Initially, based on PTX concentration, we co-cultured 4T1 cells with free PTX, HSA@PTX, or HSA-PMEMA@PTX at various PTX concentrations for 24 h, and the inhibition curves were obtained to calculate the half-maximal inhibitory concentration (IC_50_) (Figure S6). The results showed that both HSA@PTX (6.06 nM) and HSA-PMEMA@PTX (5.13 nM) exhibited significantly smaller IC_50_ values compared to free PTX (428 nM), with HSA-PMEMA@PTX demonstrating an IC_50_ approximately 85% of that of HSA@PTX, indicating superior antitumor activity, likely due to its enhanced cellular uptake. Furthermore, considering that HSA-PMEMA@PTX delivers more PTX per unit mass of HSA than HSA@PTX, we recalculated the IC_50_ based on HSA concentration. When co-cultured with 4T1 cells at various HSA concentrations for 24 h (Figure 7), HSA-PMEMA@PTX exhibited an IC_50_ of 0.535 μg HSA/mL, approximately 38% of the IC_50_ of HSA@PTX (1.41 μg HSA/mL). This indicates that HSA-PMEMA@PTX, with enhanced cellular uptake and PTX loading capacity, has superior antitumor efficacy compared to HSA@PTX.

### 3.5. Drug Release and In Vivo Delivery Behavior of HSA-PMEMA@PTX

**Figure 8:**
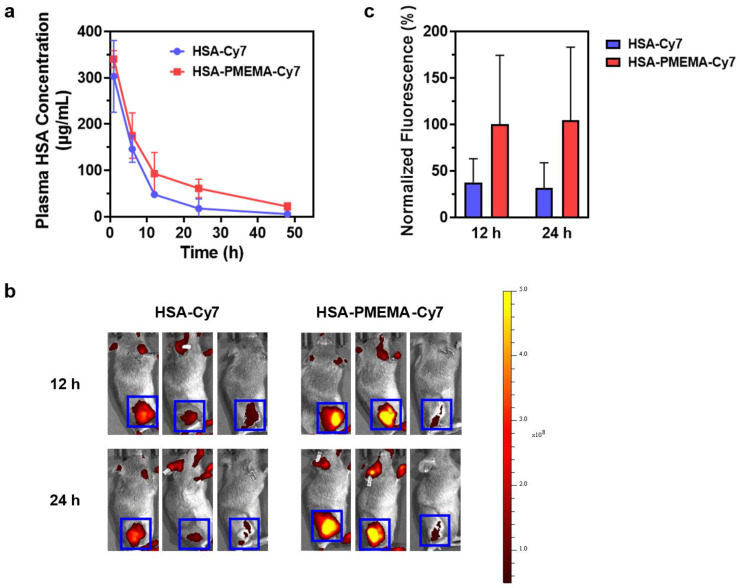
In vivo delivery behavior of HSA-PMEMA. (**a**) Time-dependent changes in plasma HSA concentration in Balb/c mice following the intravenous injection of HSA-Cy7 and HSA-PMEMA-Cy7. (**b**) Heatmap of Cy7 fluorescence intensity at the tumor site of C3H mice at 12 and 24 h after the intravenous injection of HSA-Cy7 and HSA-PMEMA-Cy7. The blue box indicates the tumor region, with the color scale on the right. (**c**) Normalized fluorescence intensity derived from panel (**b**), with the average fluorescence intensity at the tumor site of mice injected with HSA-PMEMA-Cy7 at 12 h set to 100%.

To gain a comprehensive understanding of the in vivo delivery behavior of HSA-PMEMA@PTX, we characterized its drug release profile, pharmacokinetics, and biodistribution. First, we investigated the release behavior of PTX from HSA-PMEMA@PTX using in vitro simulation. The formulation was placed in a dialysis bag and immersed in PBS at 37 °C, with the PTX concentration measured at different time points to calculate the release profile. The results, shown in Figure S7, reveal that HSA-PMEMA@PTX releases PTX more slowly than HSA@PTX after 16 h. Next, we examined the pharmacokinetics of HSA-Cy7 and HSA-PMEMA-Cy7 in Balb/c mice by fluorescently labeling the proteins with Cy7. The changes in plasma HSA concentration at various time points after injection are shown in Figure 8a. The HSA concentration in the plasma of mice injected with HSA-PMEMA-Cy7 declined more slowly than that in mice injected with HSA-Cy7, likely due to the micellar structure of HSA-PMEMA-Cy7, which reduces renal clearance. Additionally, we explored the distribution of HSA-Cy7 and HSA-PMEMA-Cy7 in tumor tissues and major organs after intravenous injection in tumor-bearing C3H mice. The results in Figure 8b,c demonstrate that, at 12 and 24 h following injection, HSA-PMEMA-Cy7 accumulates at higher levels in tumor tissues compared to HSA-Cy7, likely due to the enhanced permeability and retention (EPR) effect of the HSA-PMEMA-Cy7 nanomicelles. The half-life of HSA-Cy7 measured in this study exhibits certain discrepancies compared to that reported in the existing literature [51]. This divergence may be attributed to variations in the primary and secondary structures of HSA-Cy7 utilized in this research, as well as differences in injection dosages and detection methodologies employed in other studies. After 72 h, the levels of HSA-Cy7 and HSA-PMEMA-Cy7 in various major organs were relatively low, suggesting that the drug was efficiently cleared through metabolism (Figure S8). Given the potential toxicity of PTX, we also characterized the hemolysis of free PTX, HSA@PTX, and HSA-PMEMA@PTX in vivo. We co-cultured different concentrations of PTX, HSA@PTX, or HSA-PMEMA@PTX solutions (at typical therapeutic doses) with defibrinated sheep blood and measured the hemolysis rate (Figure S10). The results showed that the hemolysis rates of all formulations were similar to that of the negative control, indicating no significant hemolysis and demonstrating favorable safety profiles during in vivo blood circulation.

## 4. Conclusions

In this study, we successfully developed HSA-PMEMA nanomicelles via SI-PISA using HSA and MEMA, a novel hydrophobic monomer with a well-defined and stable chemical structure. The resulting HSA-PMEMA micelles exhibited a narrow size distribution and largely maintained the activity of HSA. Compared to HSA, the HSA-PMEMA micelles not only facilitated enhanced endocytosis by tumor cells, but also demonstrated superior PTX loading capacity, collectively resulting in higher intracellular PTX delivery efficiency to tumor cells. In addition to introducing a new type of hydrophobic monomer for the SI-PISA platform, this HSA-PMEMA micelle system offers a promising strategy for the development of convenient, safe, and efficient PTX delivery carriers for clinical use, with significant potential for improving the effect of cancer therapy.

## Data Availability

Data are contained within the article.

## Supplementary Materials

The following supporting information can be downloaded at: https://www.mdpi.com/article/10.3390/pharmaceutics17030316/s1, Figure S1: Complete chemical scheme of the synthesis of HSA-PMEMA; Figure S2: DLS characterization of HSA-PMEMA at different pH values (a) and concentrations (b); Figure S3: Determination of the critical micelle concentration (CMC) of HSA-PMEMA; Figure S4: Stability characterization of HSA-PMEMA in PBS and serum; Figure S5: Monitoring of the hydrolysis reaction of 4-nitrophenyl acetate catalyzed by HSA, HSA-Br, and HSA-PMEMA at 400 nm; Figure S6: Anticancer activity of HSA@PTX and HSA-PMEMA@PTX against 4T1 cells (measurement based on HSA concentration); Figure S7: Cumulative release of PTX from HSA@PTX and HSA-PMEMA@PTX in 37 °C PBS over time; Figure S8: In vivo biodistribution of the drug in tumor-bearing C3H mice at 72 h after intravenous injection of HSA-Cy7 and HSA-PMEMA-Cy7; Figure S9: Hemolysis characterization after co-culturing different concentrations of PTX, HSA@PTX, or HSA-PMEMA@PTX PBS solutions with blood; Figure S10: Standard curve of paclitaxel (PTX) in the HPLC analysis; Figure S11: HPLC results for the methanol extracts of HSA@PTX (a–c) and HSA-PMEMA@PTX (d–f); Figure S12: HPLC results for the methanol extracts in Cal27 cells co-cultured with HSA@PTX (a–c) or HSA-PMEMA@PTX (d–f). Table S1. PTX loading capacity and intracellular delivery efficiency of HSA@PTX or HSA-PMEMA@PTX based on the total mass of the delivery carrier (including polymer).
