# Supplementary material for: Novel HSA-PMEMA Nanomicelles Prepared via Site-Specific In Situ Polymerization-Induced Self-Assembly for Improved Intracellular Delivery of Paclitaxel
Source: Pharmaceutics. 2025 Mar 1;17(3):316. doi: 10.3390/pharmaceutics17030316 (PMC11945012; doi:10.3390/pharmaceutics17030316)
Supplement: Supplementary file 1 [file pharmaceutics-17-00316-s001.zip › pharmaceutics-3434785-supplementary.pdf]

pharmaceutics

MDPI

Supplementary Materials: Novel HSA-PMEMA Nanomicelles Prepared via Site-Specific In Situ Polymerization-Induced Self-Assembly for Improved Intracellular Delivery of Paclitaxel

Yang Chen, Shuang Liang, Binglin Chen, Fei Jiao, Xuliang Deng, and Xinyu Liu

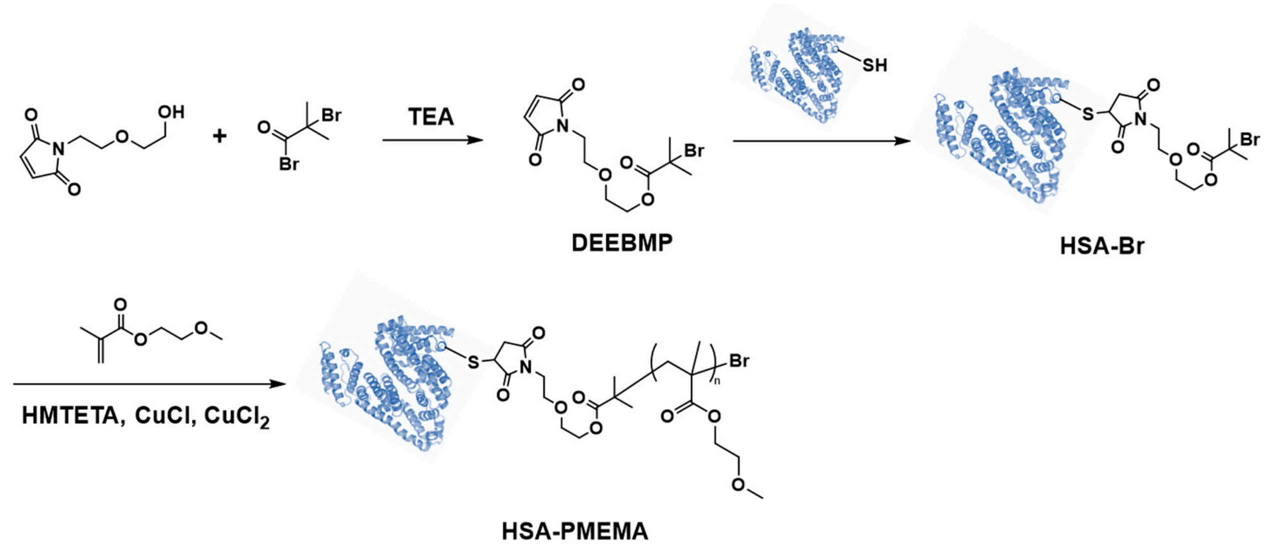

Figure S1. Complete chemical scheme of the synthesis of HSA-PMEMA.

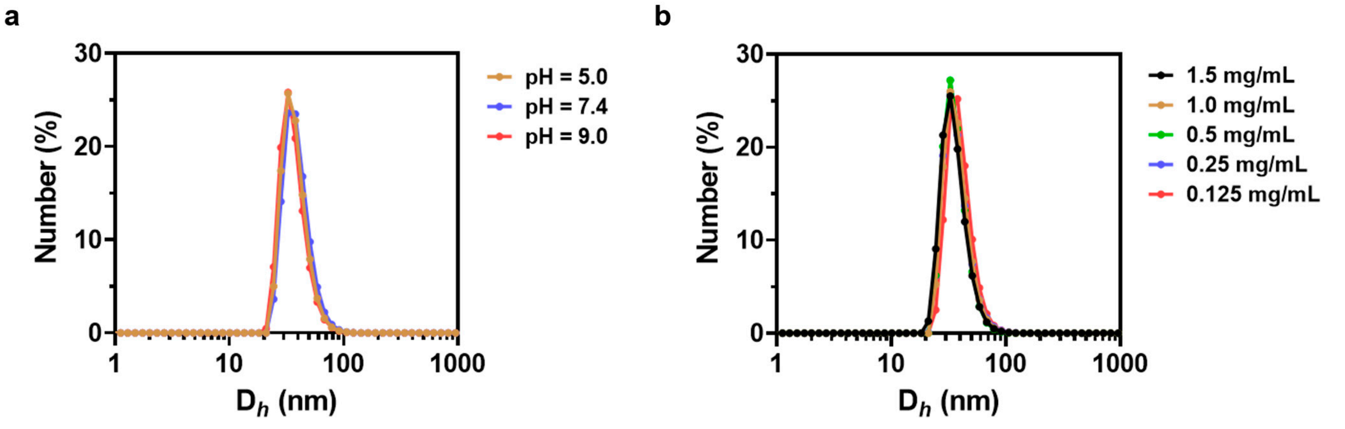

Figure S2. DLS characterization of HSA-PMEMA at different pH values (**a**) and concentrations (**b**). No significant differences in the particle size distribution of HSA-PMEMA were observed under the specified pH and concentration conditions.

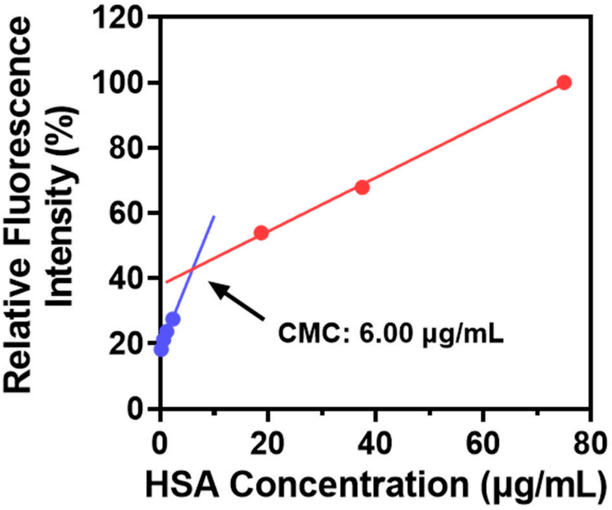

Figure S3. Determination of the critical micelle concentration (CMC) of HSA-PMEMA. The curve in the figure, formed by connecting the data points in the blue (dissociated) and red (assembled) sections, shows a distinct inflection point. The x-coordinate of the intersection of the fitted lines for each data set represents the CMC.

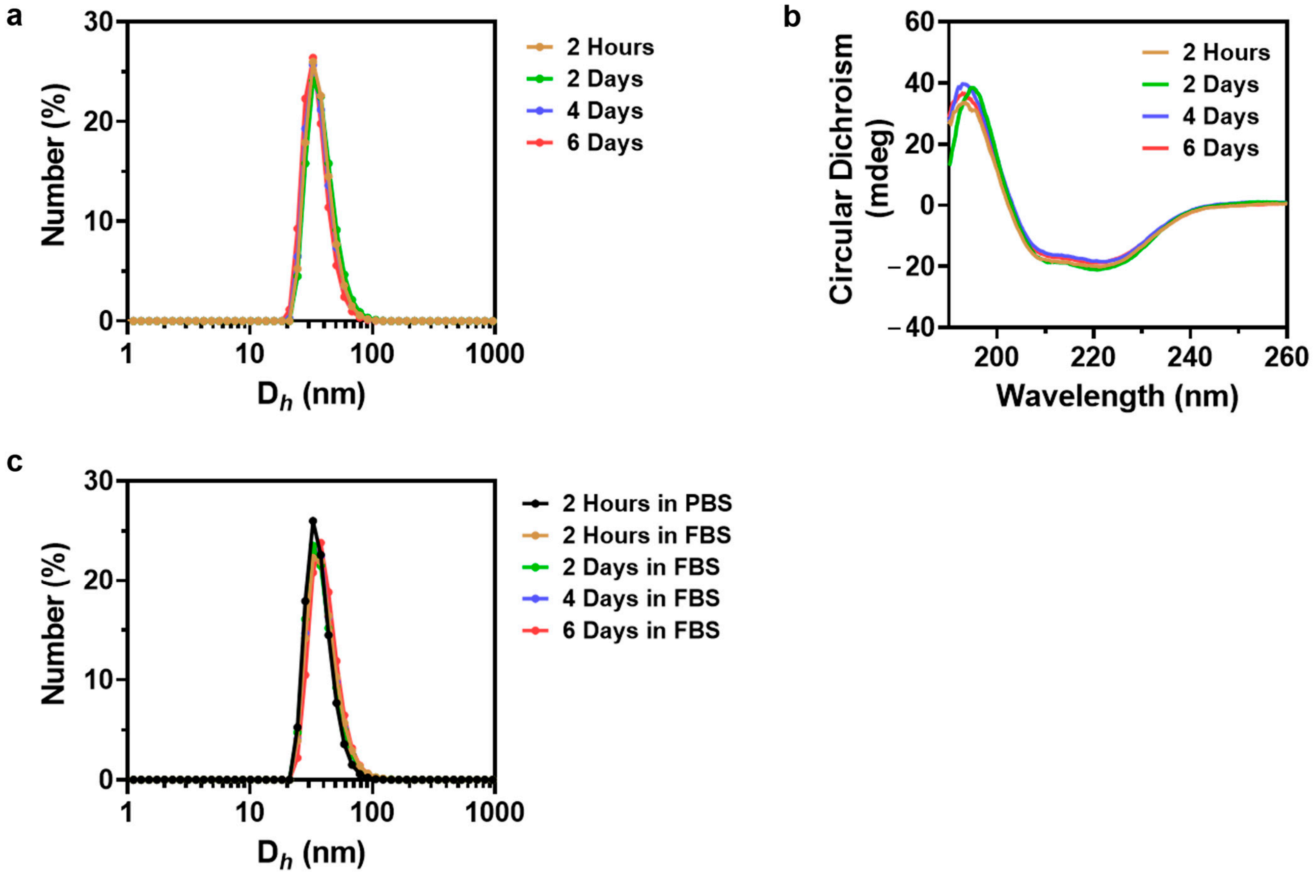

Figure S4. Stability characterization of HSA-PMEMA in PBS and serum. (**a**) DLS characterization of HSA-PMEMA after different storage times in PBS. (**b**) CD characterization of HSA-PMEMA after different storage times in PBS. (**c**) DLS characterization of HSA-PMEMA after incubation with fetal bovine serum (FBS) for different durations. Under the conditions tested, no significant changes in particle size distribution or CD spectra were observed, indicating that HSA-PMEMA is stable during storage both in PBS and in serum.

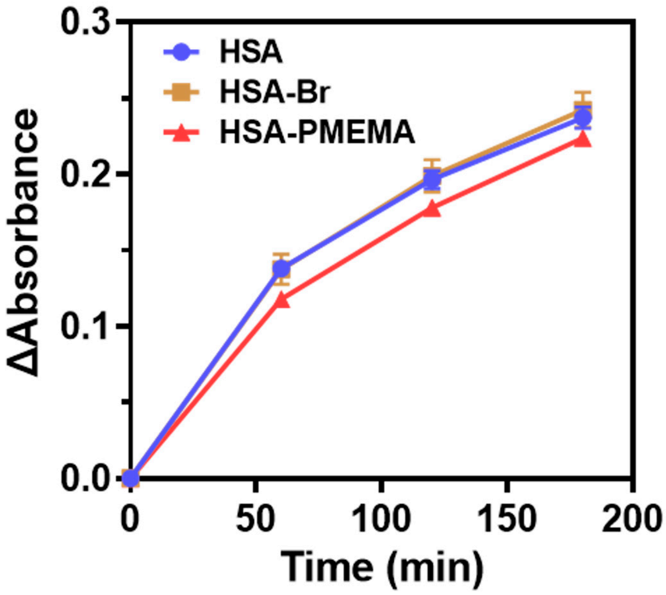

Figure S5. Monitoring of the hydrolysis reaction of 4-nitrophenyl acetate catalyzed by HSA, HSA-Br, and HSA-PMEMA at 400 nm. ΔAbsorbance is compared to the start of the reaction, and the absorbance of the background is subtracted.

Table S1. PTX loading capacity and intracellular delivery efficiency of HSA@PTX or HSA-PMEMA@PTX based on the total mass of the delivery carrier (including polymer).

Parameters	HSA@PTX	HSA-PMEMA@PTX
PTX loading content (%)	0.368 ± 0.003	0.370 ± 0.002
Normalized PTX loading content * (%)	100 ± 1	101 ± 1
PTX loading efficiency (%)	2.85 ± 0.02	4.06 ± 0.03
Normalized PTX loading content * (%)	100 ± 1	143 ± 1
Intracellular PTX delivery efficiency (%)	0.0064 ± 0.0002	0.0081 ± 0.0001
Normalized intracellular PTX delivery efficiency * (%)	100 ± 3	125 ± 2

* The normalized parameters of HSA@PTX are set as 100%.

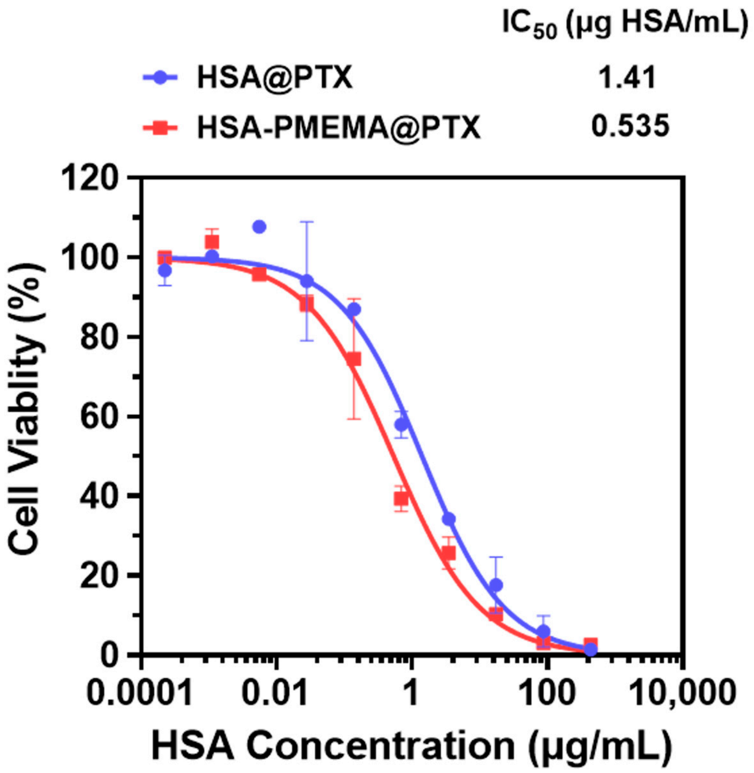

Figure S6. Anticancer activity of HSA@PTX and HSA-PMEMA@PTX against 4T1 cells (measurement based on HSA concentration). The half-maximal inhibitory concentration (IC_{50}) was calculated from the inhibition curves fitted.

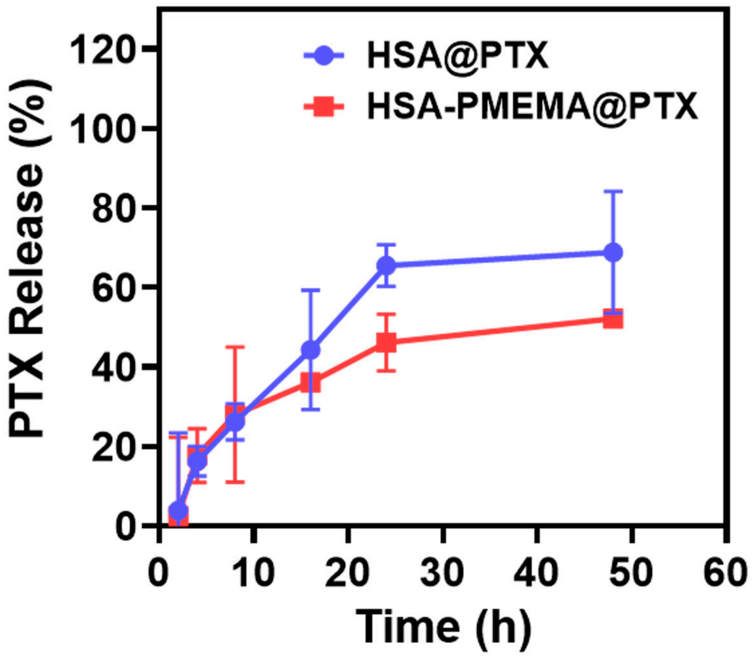

Figure S7. Cumulative release of PTX from HSA@PTX and HSA-PMEMA@PTX in 37°C PBS over time. The total PTX release is set as 100% of the respective PTX load in each formulation.

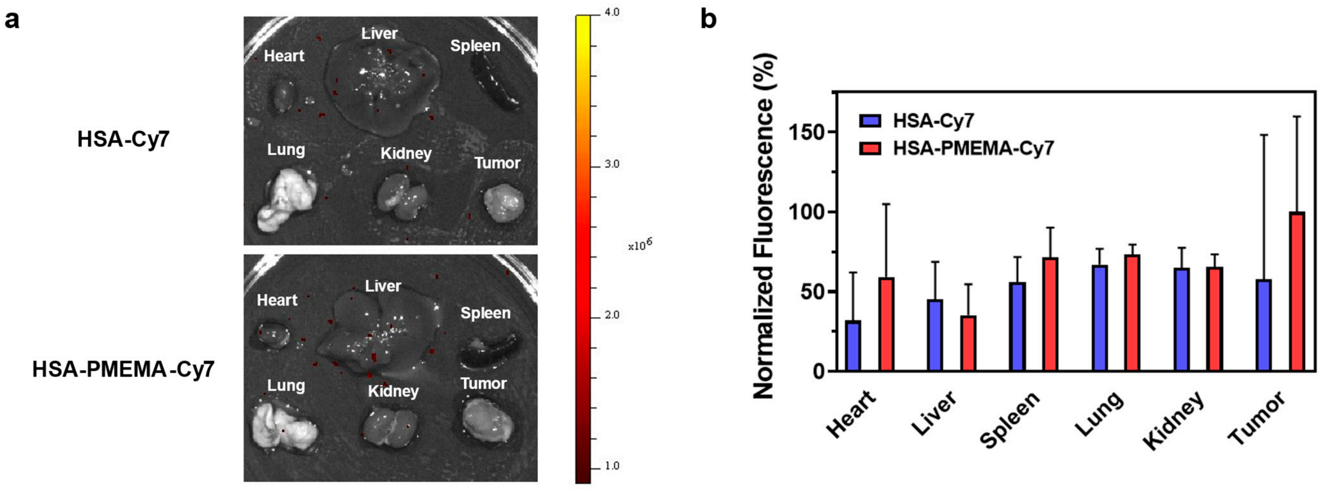

Figure S8. In vivo biodistribution of the drug in tumor-bearing C3H mice at 72 hours after intravenous injection of HSA-Cy7 and HSA-PMEMA-Cy7. (**a**) Heatmap of Cy7 fluorescence intensity in major organs and the tumor in mice at 72 hours after injection. (**b**) Normalized fluorescence intensity derived from panel (**a**), with the average fluorescence intensity in the tumors of mice injected with HSA-PMEMA-Cy7 set to 100%.

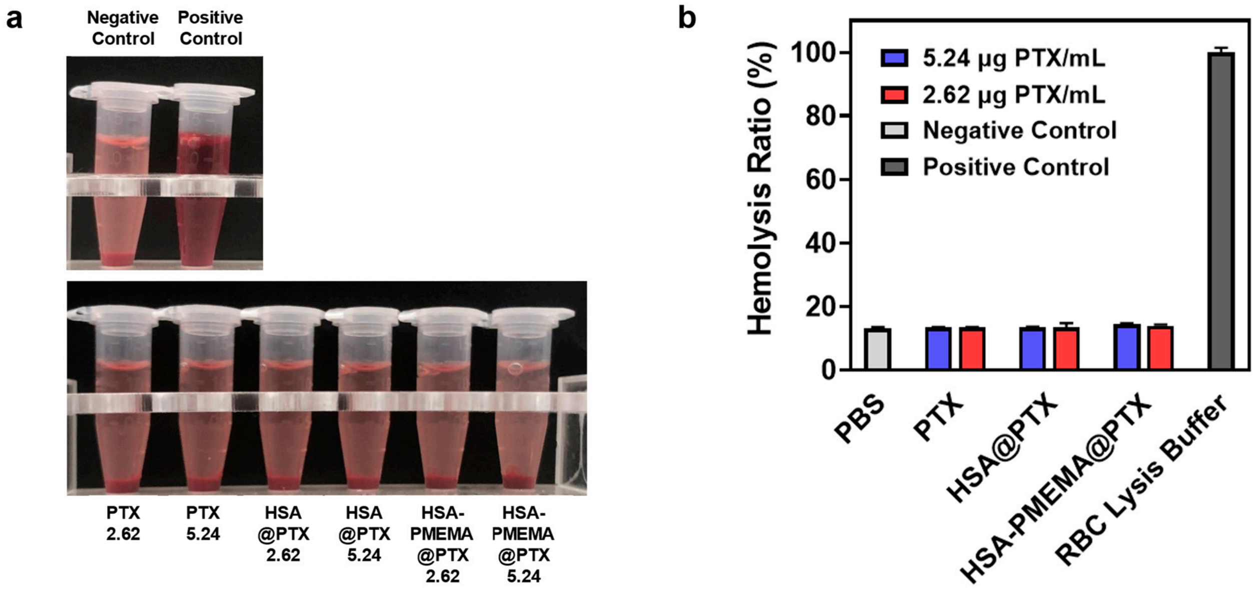

Figure S9. Hemolysis characterization after co-culturing different concentrations of PTX, HSA@PTX, or HSA-PMEMA@PTX PBS solutions with blood. (**a**) Appearance of serum after centrifugation following co-culturing each solution with blood. (**b**) Hemolysis ratio measurement for the different groups. The hemolysis ratio in each group was similar to that of the negative control, indicating no significant hemolysis for any of the formulations.

a

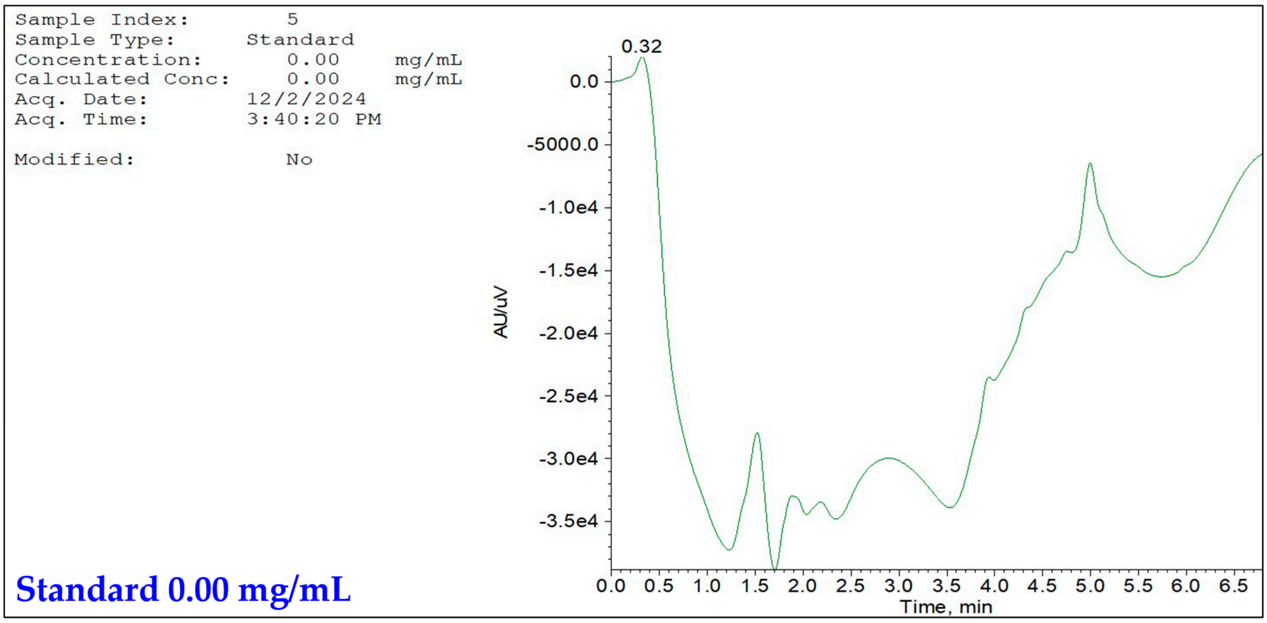
Sample Index: 5
Sample Type: Standard
Concentration: 0.00 mg/mL
Calculated Conc: 0.00 mg/mL
Acq. Date: 12/2/2024
Acq. Time: 3:40:20 PM
Modified: No
0.32
AU/uV
Time, min
Standard 0.00 mg/mL

b

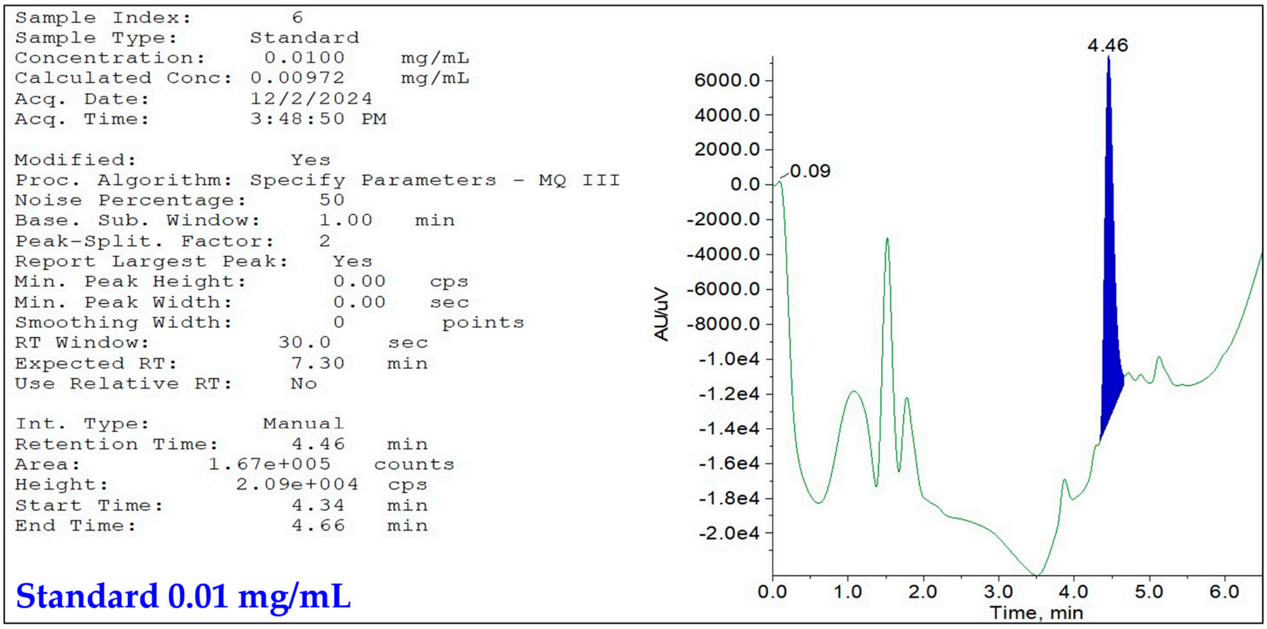
Sample Index: 6
Sample Type: Standard
Concentration: 0.0100 mg/mL
Calculated Conc: 0.00972 mg/mL
Acq. Date: 12/2/2024
Acq. Time: 3:48:50 PM
Modified: Yes
Proc. Algorithm: Specify Parameters - MQ III
Noise Percentage: 50
Base. Sub. Window: 1.00 min
Peak-Split. Factor: 2
Report Largest Peak: Yes
Min. Peak Height: 0.00 cps
Min. Peak Width: 0.00 sec
Smoothing Width: 0 points
RT Window: 30.0 sec
Expected RT: 7.30 min
Use Relative RT: No
Int. Type: Manual
Retention Time: 4.46 min
Area: 1.67e+005 counts
Height: 2.09e+004 cps
Start Time: 4.34 min
End Time: 4.66 min
4.46
0.09
AU/uV
Time, min
Standard 0.01 mg/mL

c

Sample Index: 7
Sample Type: Standard
Concentration: 0.0500 mg/mL
Calculated Conc: 0.0506 mg/mL
Acq. Date: 12/2/2024
Acq. Time: 3:57:26 PM

Modified: Yes
Proc. Algorithm: Specify Parameters - MQ III
Noise Percentage: 50
Base. Sub. Window: 1.00 min
Peak-Split. Factor: 2
Report Largest Peak: Yes
Min. Peak Height: 0.00 cps
Min. Peak Width: 0.00 sec
Smoothing Width: 0 points
RT Window: 30.0 sec
Expected RT: 7.30 min
Use Relative RT: No

Int. Type: Manual
Retention Time: 4.44 min
Area: 8.69e+005 counts
Height: 1.10e+005 cps
Start Time: 4.33 min
End Time: 4.65 min

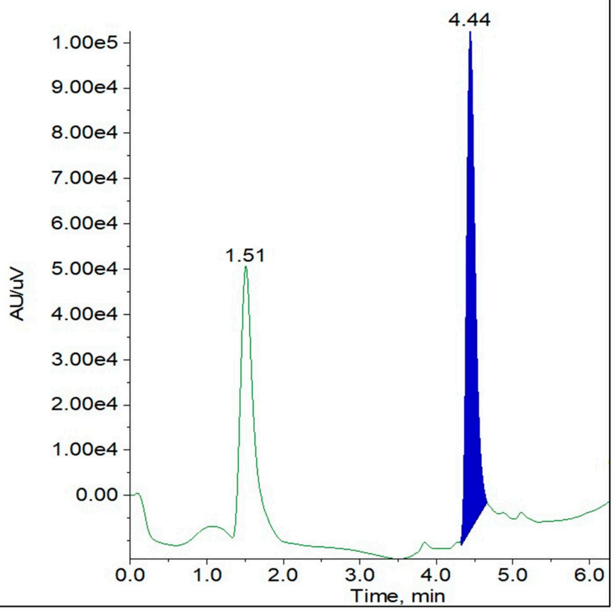

Standard 0.05 mg/mL

d

Sample Index: 8
Sample Type: Standard
Concentration: 0.100 mg/mL
Calculated Conc: 0.0894 mg/mL
Acq. Date: 12/2/2024
Acq. Time: 4:05:56 PM

Modified: Yes
Proc. Algorithm: Specify Parameters - MQ III
Noise Percentage: 50
Base. Sub. Window: 1.00 min
Peak-Split. Factor: 2
Report Largest Peak: Yes
Min. Peak Height: 0.00 cps
Min. Peak Width: 0.00 sec
Smoothing Width: 0 points
RT Window: 30.0 sec
Expected RT: 7.30 min
Use Relative RT: No

Int. Type: Manual
Retention Time: 4.43 min
Area: 1.54e+006 counts
Height: 1.80e+005 cps
Start Time: 4.29 min
End Time: 4.79 min

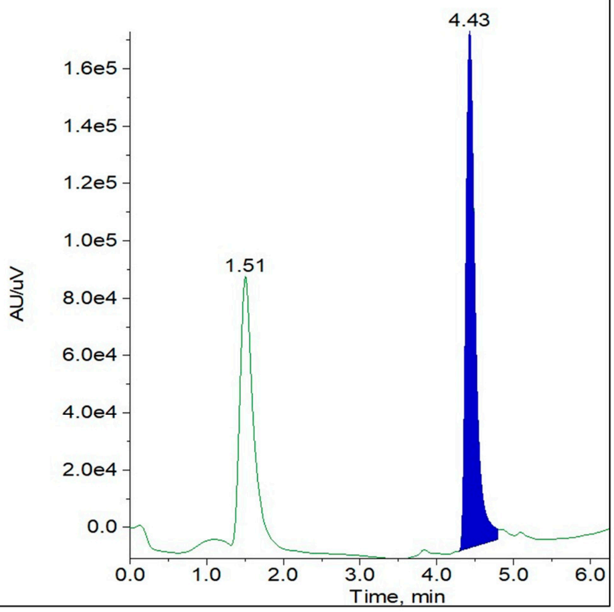

Standard 0.10 mg/mL

e

```
Sample Index:        9
Sample Type:      Standard
Concentration:      0.500     mg/mL
Calculated Conc:    0.579     mg/mL
Acq. Date:        12/2/2024
Acq. Time:        4:14:28 PM

Modified:               Yes
Proc. Algorithm: Specify Parameters - MQ III
Noise Percentage:       50
Base. Sub. Window:     1.00    min
Peak-Split. Factor:    2
Report Largest Peak:    Yes
Min. Peak Height:       0.00    cps
Min. Peak Width:        0.00    sec
Smoothing Width:        0          points
RT Window:            30.0     sec
Expected RT:            7.30    min
Use Relative RT:        No

Int. Type:             Manual
Retention Time:         4.44    min
Area:             9.95e+006   counts
Height:              1.11e+006   cps
Start Time:             4.33    min
End Time:               5.04    min
```

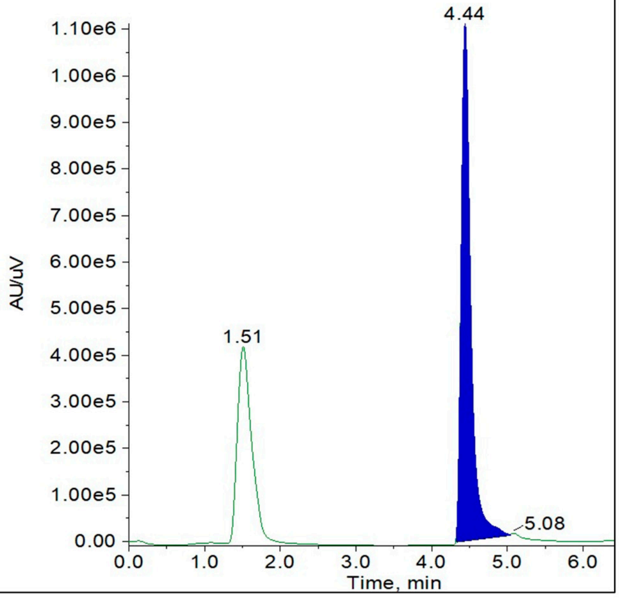

Standard 0.50 mg/mL

f

```
Sample Index:       10
Sample Type:      Standard
Concentration:      1.00      mg/mL
Calculated Conc:    0.965     mg/mL
Acq. Date:        12/2/2024
Acq. Time:        4:23:04 PM

Modified:               Yes
Proc. Algorithm: Specify Parameters - MQ III
Noise Percentage:       50
Base. Sub. Window:     1.00    min
Peak-Split. Factor:    2
Report Largest Peak:    Yes
Min. Peak Height:       0.00    cps
Min. Peak Width:        0.00    sec
Smoothing Width:        0          points
RT Window:            30.0     sec
Expected RT:            7.30    min
Use Relative RT:        No

Int. Type:             Manual
Retention Time:         4.45    min
Area:             1.66e+007   counts
Height:              1.73e+006   cps
Start Time:             4.31    min
End Time:               5.21    min
```

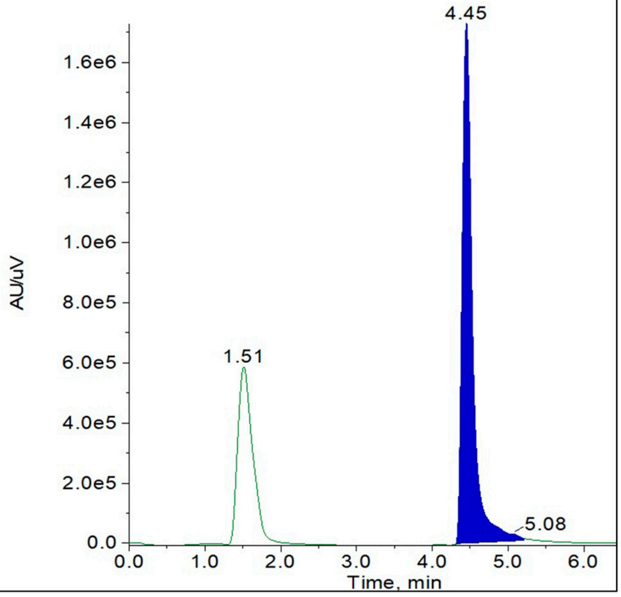

Standard 1.00 mg/mL

g

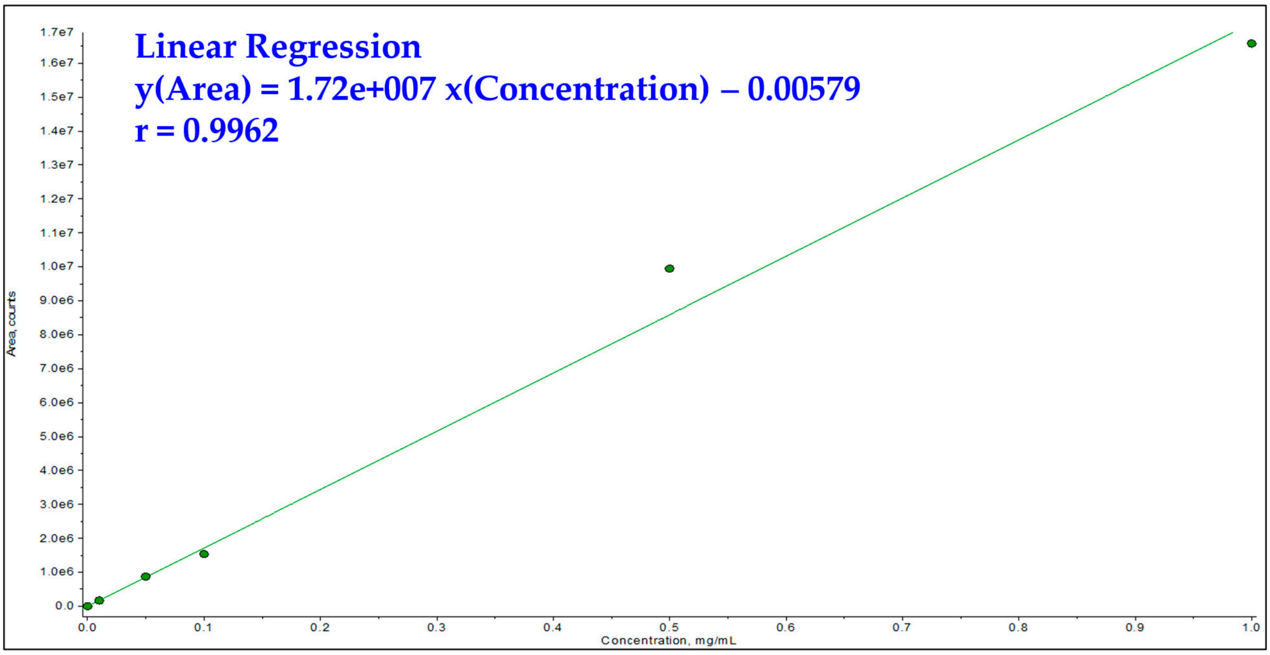

Figure S10. Standard curve of paclitaxel (PTX) in the HPLC analysis. (**a**–**f**) The HPLC result of the standard samples at concentrations of 0.00, 0.01, 0.05, 0.10, 0.50, and 1.00 mg/mL, respectively. (**g**) Standard curve of PTX.

a

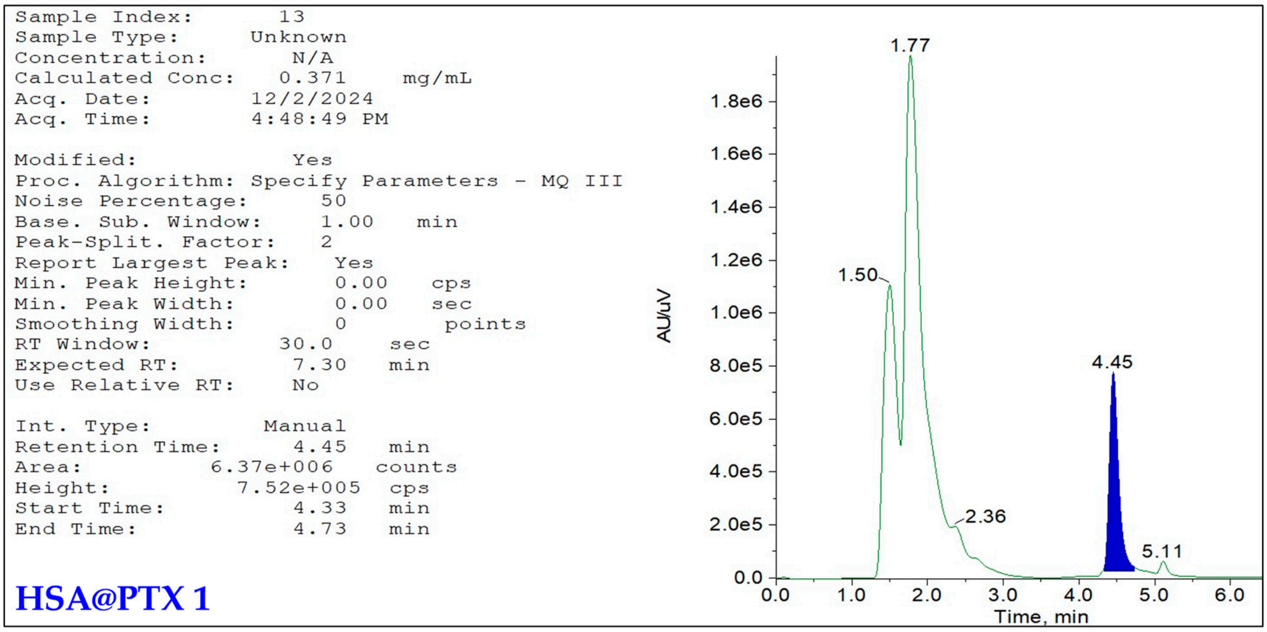

b

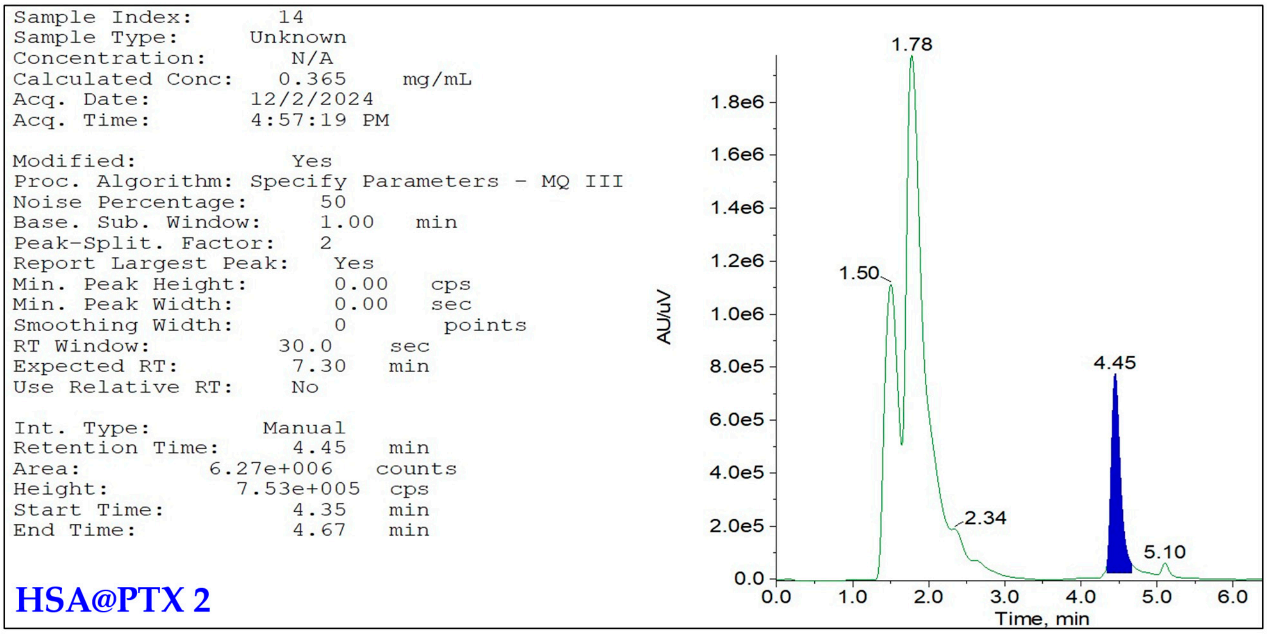
Sample Index: 14
Sample Type: Unknown
Concentration: N/A
Calculated Conc: 0.365 mg/mL
Acq. Date: 12/2/2024
Acq. Time: 4:57:19 PM
Modified: Yes
Proc. Algorithm: Specify Parameters - MQ III
Noise Percentage: 50
Base. Sub. Window: 1.00 min
Peak-Split. Factor: 2
Report Largest Peak: Yes
Min. Peak Height: 0.00 cps
Min. Peak Width: 0.00 sec
Smoothing Width: 0 points
RT Window: 30.0 sec
Expected RT: 7.30 min
Use Relative RT: No
Int. Type: Manual
Retention Time: 4.45 min
Area: 6.27e+006 counts
Height: 7.53e+005 cps
Start Time: 4.35 min
End Time: 4.67 min
HSA@PTX 2
AU/uV
Time, min
1.50
1.78
2.34
4.45
5.10

c

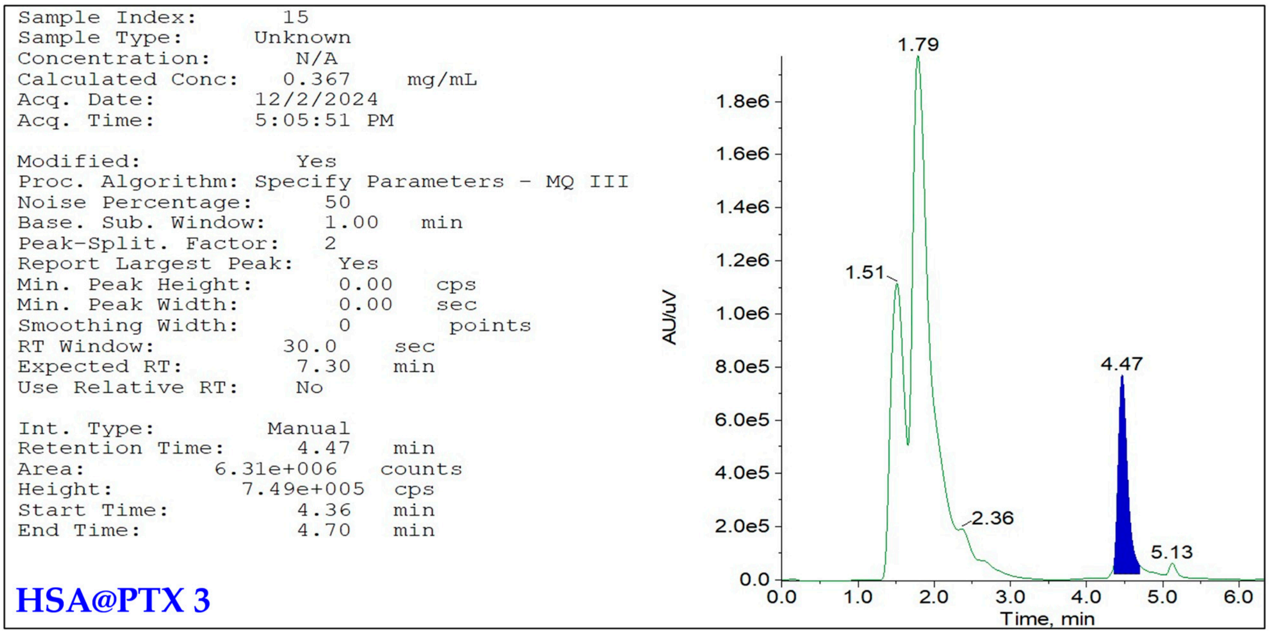
Sample Index: 15
Sample Type: Unknown
Concentration: N/A
Calculated Conc: 0.367 mg/mL
Acq. Date: 12/2/2024
Acq. Time: 5:05:51 PM
Modified: Yes
Proc. Algorithm: Specify Parameters - MQ III
Noise Percentage: 50
Base. Sub. Window: 1.00 min
Peak-Split. Factor: 2
Report Largest Peak: Yes
Min. Peak Height: 0.00 cps
Min. Peak Width: 0.00 sec
Smoothing Width: 0 points
RT Window: 30.0 sec
Expected RT: 7.30 min
Use Relative RT: No
Int. Type: Manual
Retention Time: 4.47 min
Area: 6.31e+006 counts
Height: 7.49e+005 cps
Start Time: 4.36 min
End Time: 4.70 min
HSA@PTX 3
AU/uV
Time, min
1.51
1.79
2.36
4.47
5.13

d

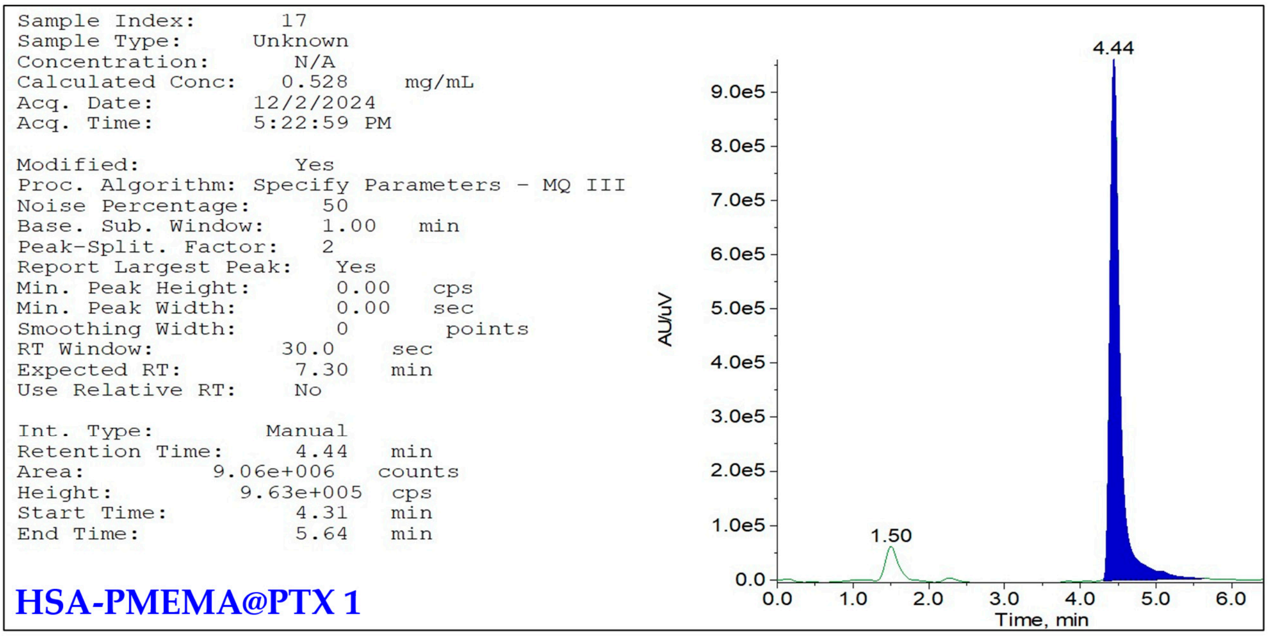
Sample Index: 17
Sample Type: Unknown
Concentration: N/A
Calculated Conc: 0.528 mg/mL
Acq. Date: 12/2/2024
Acq. Time: 5:22:59 PM
Modified: Yes
Proc. Algorithm: Specify Parameters - MQ III
Noise Percentage: 50
Base. Sub. Window: 1.00 min
Peak-Split. Factor: 2
Report Largest Peak: Yes
Min. Peak Height: 0.00 cps
Min. Peak Width: 0.00 sec
Smoothing Width: 0 points
RT Window: 30.0 sec
Expected RT: 7.30 min
Use Relative RT: No
Int. Type: Manual
Retention Time: 4.44 min
Area: 9.06e+006 counts
Height: 9.63e+005 cps
Start Time: 4.31 min
End Time: 5.64 min
HSA-PMEMA@PTX 1
AU/uV
Time, min
4.44
1.50
9.0e5
8.0e5
7.0e5
6.0e5
5.0e5
4.0e5
3.0e5
2.0e5
1.0e5
0.0
0.0 1.0 2.0 3.0 4.0 5.0 6.0

e

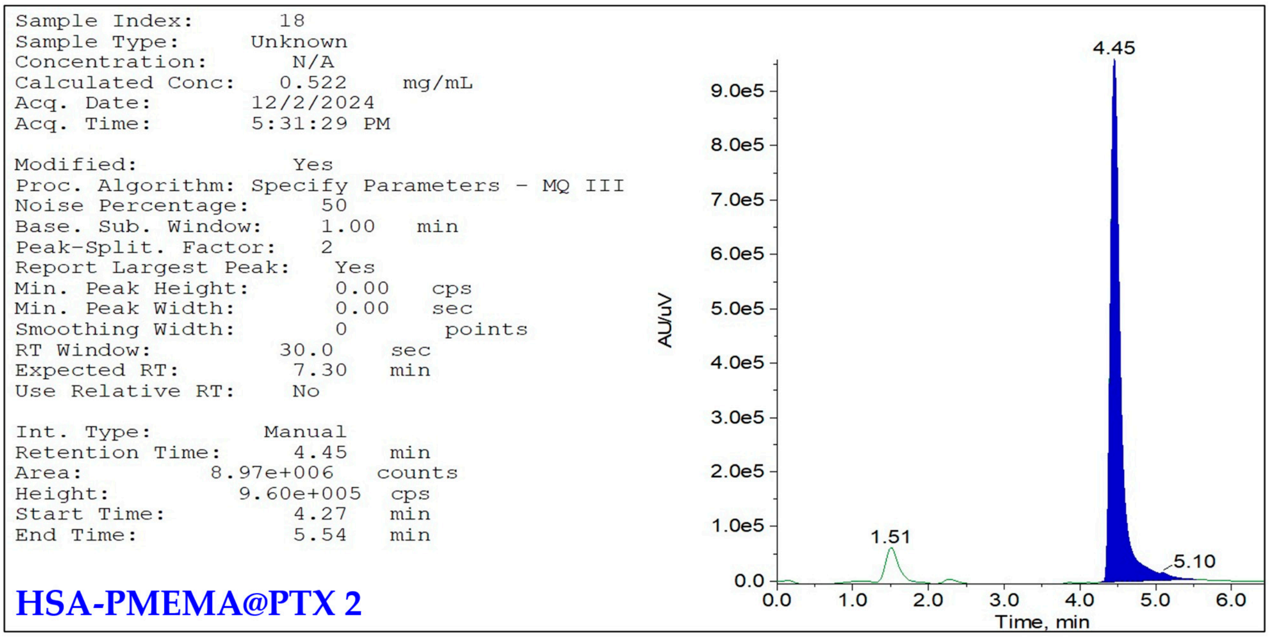
Sample Index: 18
Sample Type: Unknown
Concentration: N/A
Calculated Conc: 0.522 mg/mL
Acq. Date: 12/2/2024
Acq. Time: 5:31:29 PM
Modified: Yes
Proc. Algorithm: Specify Parameters - MQ III
Noise Percentage: 50
Base. Sub. Window: 1.00 min
Peak-Split. Factor: 2
Report Largest Peak: Yes
Min. Peak Height: 0.00 cps
Min. Peak Width: 0.00 sec
Smoothing Width: 0 points
RT Window: 30.0 sec
Expected RT: 7.30 min
Use Relative RT: No
Int. Type: Manual
Retention Time: 4.45 min
Area: 8.97e+006 counts
Height: 9.60e+005 cps
Start Time: 4.27 min
End Time: 5.54 min
HSA-PMEMA@PTX 2
AU/uV
Time, min
4.45
1.51
5.10
9.0e5
8.0e5
7.0e5
6.0e5
5.0e5
4.0e5
3.0e5
2.0e5
1.0e5
0.0
0.0 1.0 2.0 3.0 4.0 5.0 6.0

f

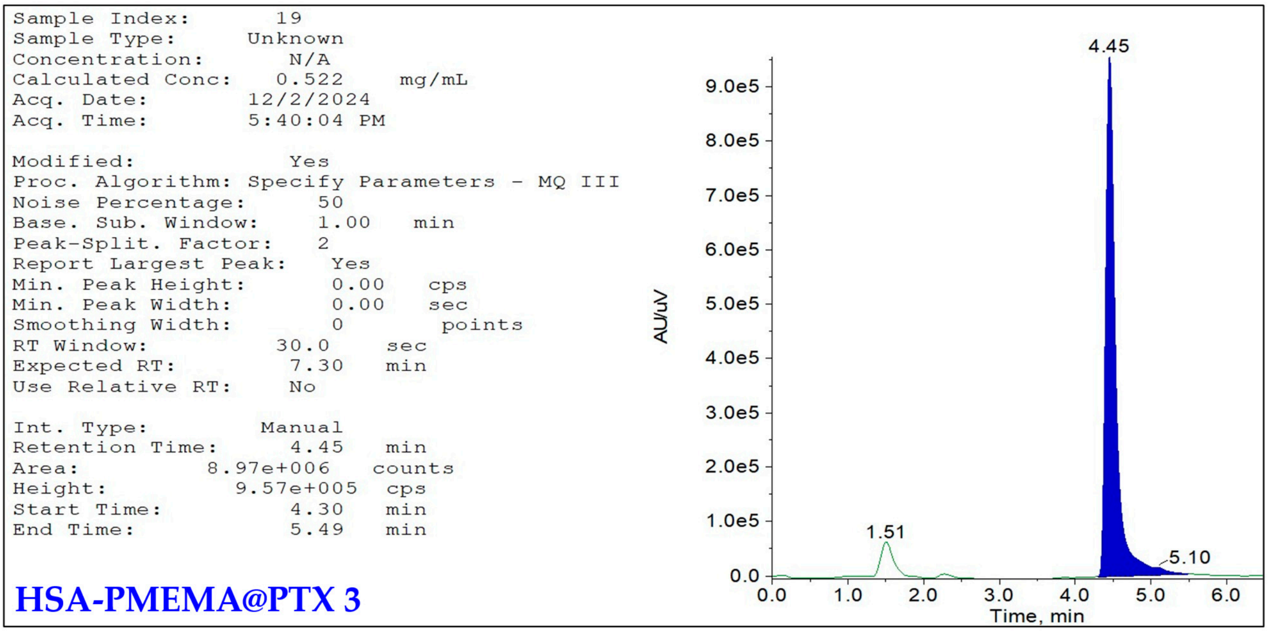

Figure S11. HPLC results for the methanol extracts of HSA@PTX (**a**–**c**) and HSA-PMEMA@PTX (**d**–**f**).

a

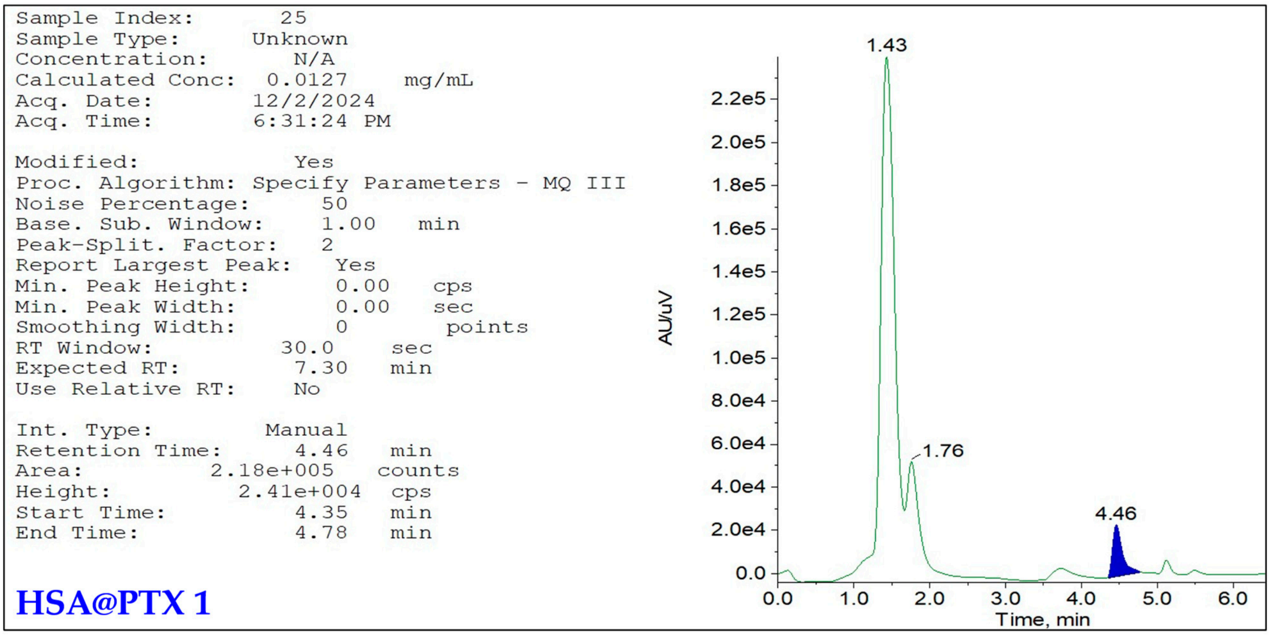

b

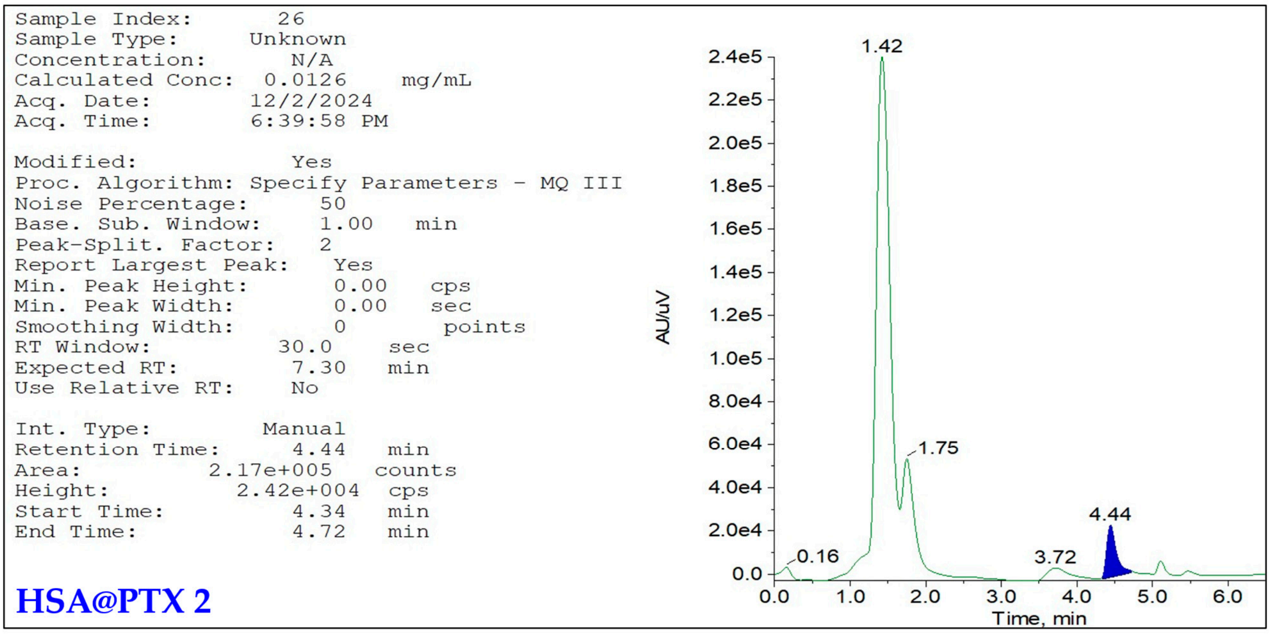
Sample Index: 26
Sample Type: Unknown
Concentration: N/A
Calculated Conc: 0.0126 mg/mL
Acq. Date: 12/2/2024
Acq. Time: 6:39:58 PM
Modified: Yes
Proc. Algorithm: Specify Parameters - MQ III
Noise Percentage: 50
Base. Sub. Window: 1.00 min
Peak-Split. Factor: 2
Report Largest Peak: Yes
Min. Peak Height: 0.00 cps
Min. Peak Width: 0.00 sec
Smoothing Width: 0 points
RT Window: 30.0 sec
Expected RT: 7.30 min
Use Relative RT: No
Int. Type: Manual
Retention Time: 4.44 min
Area: 2.17e+005 counts
Height: 2.42e+004 cps
Start Time: 4.34 min
End Time: 4.72 min
HSA@PTX 2
AU/uV
2.4e5
2.2e5
2.0e5
1.8e5
1.6e5
1.4e5
1.2e5
1.0e5
8.0e4
6.0e4
4.0e4
2.0e4
0.0
1.42
1.75
0.16
3.72
4.44
0.0
1.0
2.0
3.0
4.0
5.0
6.0
Time, min

c

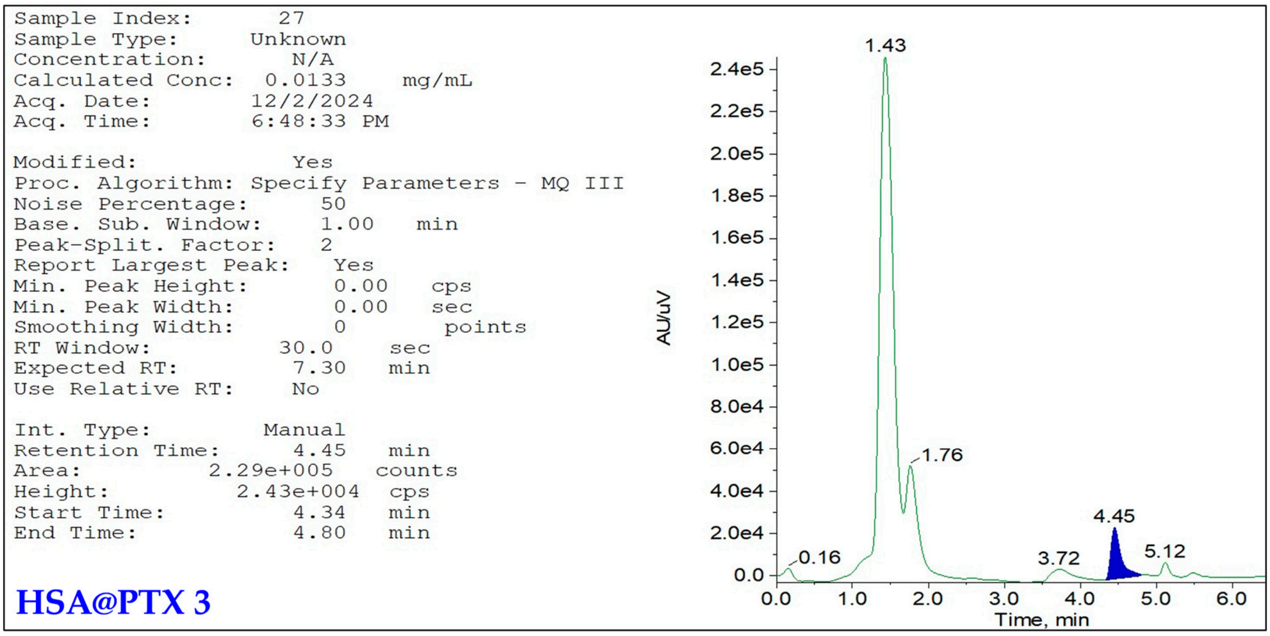
Sample Index: 27
Sample Type: Unknown
Concentration: N/A
Calculated Conc: 0.0133 mg/mL
Acq. Date: 12/2/2024
Acq. Time: 6:48:33 PM
Modified: Yes
Proc. Algorithm: Specify Parameters - MQ III
Noise Percentage: 50
Base. Sub. Window: 1.00 min
Peak-Split. Factor: 2
Report Largest Peak: Yes
Min. Peak Height: 0.00 cps
Min. Peak Width: 0.00 sec
Smoothing Width: 0 points
RT Window: 30.0 sec
Expected RT: 7.30 min
Use Relative RT: No
Int. Type: Manual
Retention Time: 4.45 min
Area: 2.29e+005 counts
Height: 2.43e+004 cps
Start Time: 4.34 min
End Time: 4.80 min
HSA@PTX 3
AU/uV
2.4e5
2.2e5
2.0e5
1.8e5
1.6e5
1.4e5
1.2e5
1.0e5
8.0e4
6.0e4
4.0e4
2.0e4
0.0
1.43
1.76
0.16
3.72
4.45
5.12
0.0
1.0
2.0
3.0
4.0
5.0
6.0
Time, min

d

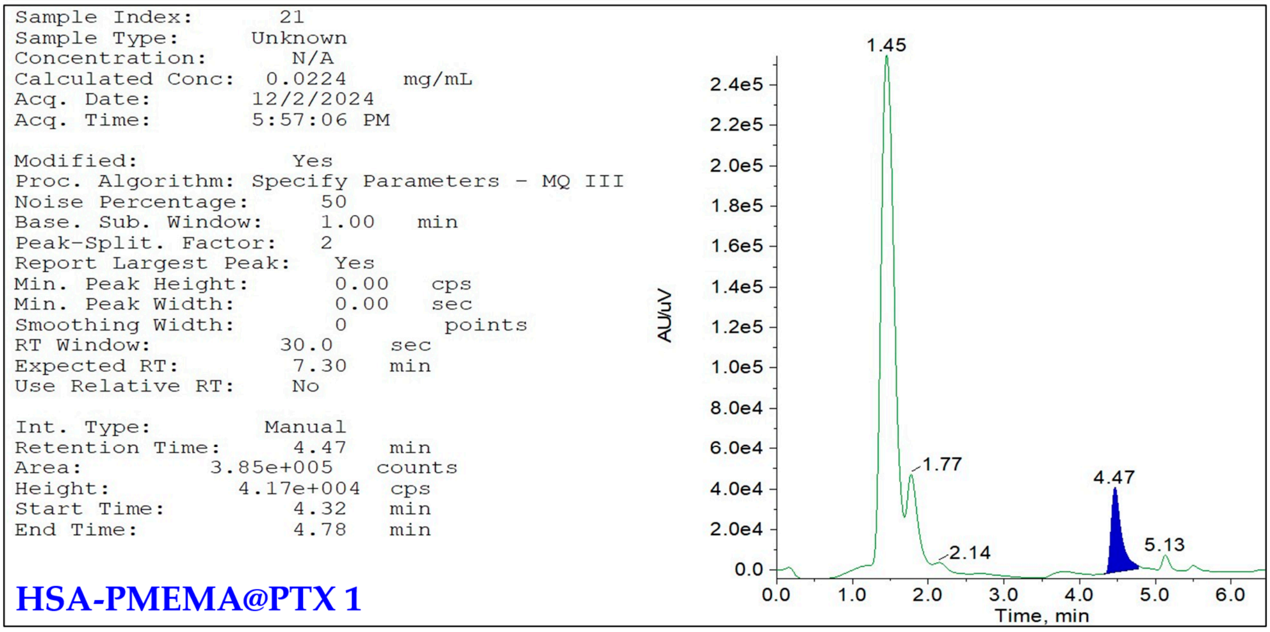
Sample Index: 21
Sample Type: Unknown
Concentration: N/A
Calculated Conc: 0.0224 mg/mL
Acq. Date: 12/2/2024
Acq. Time: 5:57:06 PM
Modified: Yes
Proc. Algorithm: Specify Parameters - MQ III
Noise Percentage: 50
Base. Sub. Window: 1.00 min
Peak-Split. Factor: 2
Report Largest Peak: Yes
Min. Peak Height: 0.00 cps
Min. Peak Width: 0.00 sec
Smoothing Width: 0 points
RT Window: 30.0 sec
Expected RT: 7.30 min
Use Relative RT: No
Int. Type: Manual
Retention Time: 4.47 min
Area: 3.85e+005 counts
Height: 4.17e+004 cps
Start Time: 4.32 min
End Time: 4.78 min
HSA-PMEMA@PTX 1
1.45
1.77
2.14
4.47
5.13
AU/uV
2.4e5
2.2e5
2.0e5
1.8e5
1.6e5
1.4e5
1.2e5
1.0e5
8.0e4
6.0e4
4.0e4
2.0e4
0.0
0.0
1.0
2.0
3.0
4.0
5.0
6.0
Time, min

e

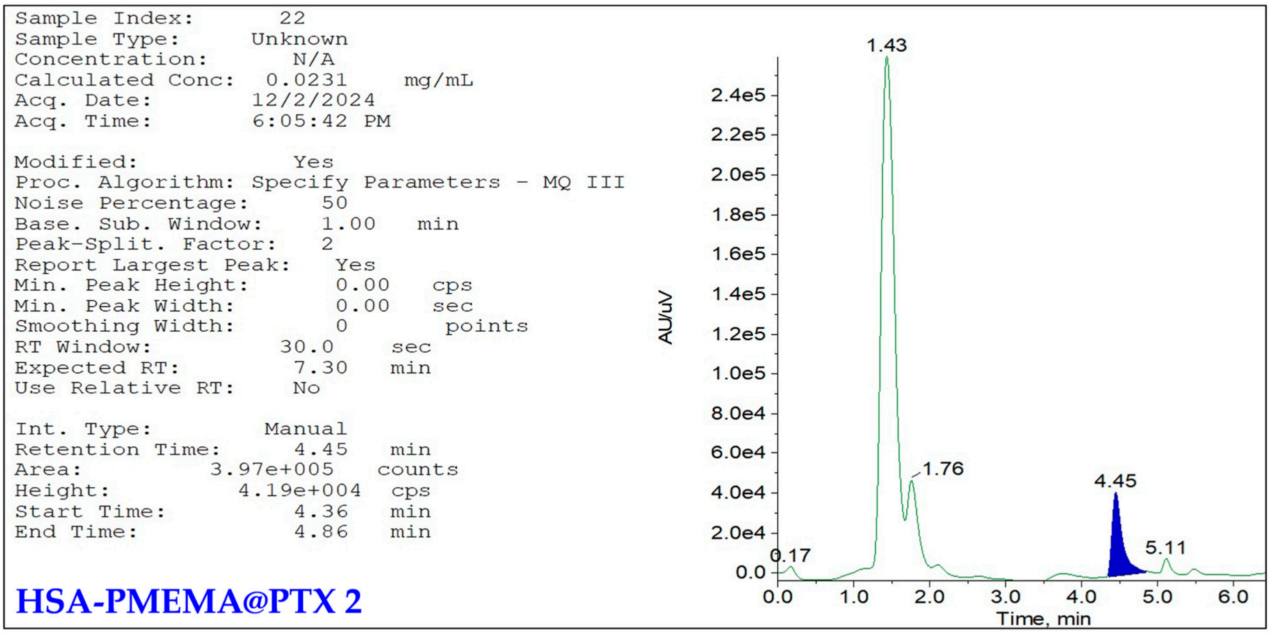
Sample Index: 22
Sample Type: Unknown
Concentration: N/A
Calculated Conc: 0.0231 mg/mL
Acq. Date: 12/2/2024
Acq. Time: 6:05:42 PM
Modified: Yes
Proc. Algorithm: Specify Parameters - MQ III
Noise Percentage: 50
Base. Sub. Window: 1.00 min
Peak-Split. Factor: 2
Report Largest Peak: Yes
Min. Peak Height: 0.00 cps
Min. Peak Width: 0.00 sec
Smoothing Width: 0 points
RT Window: 30.0 sec
Expected RT: 7.30 min
Use Relative RT: No
Int. Type: Manual
Retention Time: 4.45 min
Area: 3.97e+005 counts
Height: 4.19e+004 cps
Start Time: 4.36 min
End Time: 4.86 min
HSA-PMEMA@PTX 2
1.43
1.76
0.17
4.45
5.11
AU/uV
2.4e5
2.2e5
2.0e5
1.8e5
1.6e5
1.4e5
1.2e5
1.0e5
8.0e4
6.0e4
4.0e4
2.0e4
0.0
0.0
1.0
2.0
3.0
4.0
5.0
6.0
Time, min

f

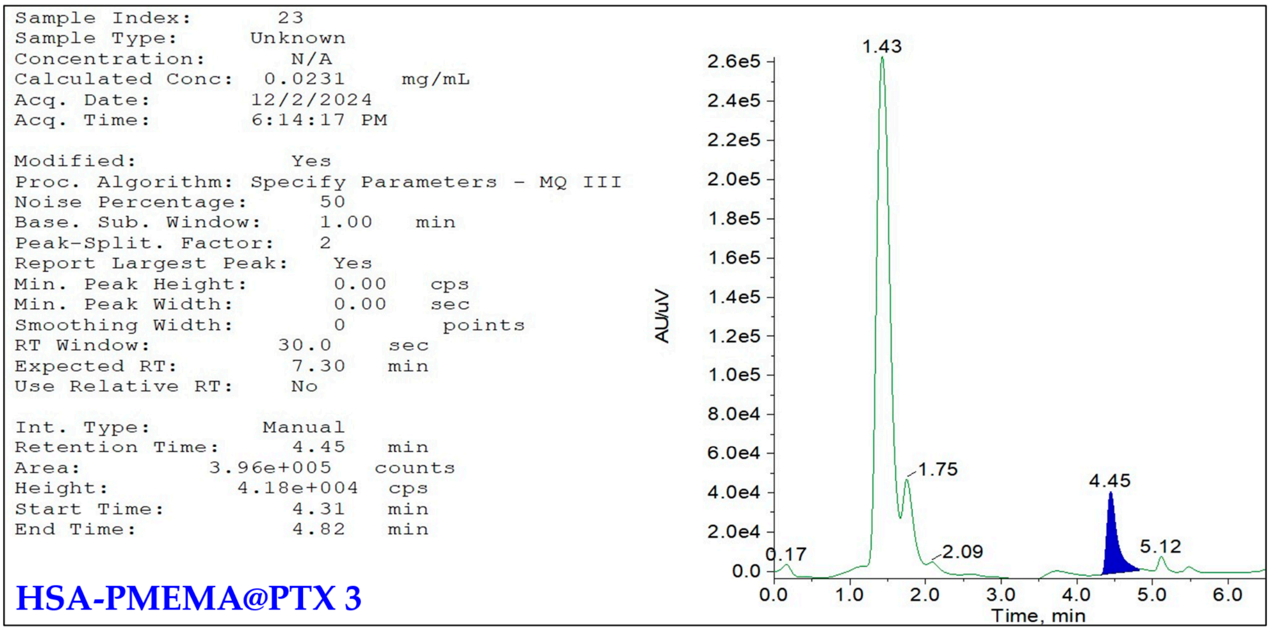

Figure S12. HPLC results for the methanol extracts in Cal27 cells co-cultured with HSA@PTX (**a–c**) or HSA-PMEMA@PTX (**d–f**).
